# Establishing a natural history of X-linked dystonia parkinsonism

**DOI:** 10.1093/braincomms/fcad106

**Published:** 2023-04-04

**Authors:** Patrick Acuna, Melanie Leigh Supnet-Wells, Neil A Spencer, Jan Kristoper de Guzman, Massimiliano Russo, Ann Hunt, Christopher Stephen, Criscely Go, Samuel Carr, Niecy Grace Ganza, John Benedict Lagarde, Shin Begalan, Trisha Multhaupt-Buell, Gabrielle Aldykiewicz, Lisa Paul, Laurie Ozelius, D Cristopher Bragg, Bridget Perry, Jordan R Green, Jeffrey W Miller, Nutan Sharma

**Affiliations:** Department of Neurology, Massachusetts General Hospital and Harvard Medical School, Boston, MA 02114, USA; The Collaborative Center for X-linked Dystonia-Parkinsonism, Massachusetts General Hospital, Charlestown, MA 02129, USA; Sunshine Care Foundation, The Health Centrum, Roxas City, Capiz 5800Philippines; Department of Neurology, Massachusetts General Hospital and Harvard Medical School, Boston, MA 02114, USA; The Collaborative Center for X-linked Dystonia-Parkinsonism, Massachusetts General Hospital, Charlestown, MA 02129, USA; Department of Statistics, University of Connecticut, Storrs, CT 06269, USA; Department of Neurology, Jose Reyes Memorial Medical Center, Manila, Metro Manila, 1012Philippines; Sunshine Care Foundation, The Health Centrum, Roxas City, Capiz 5800Philippines; Division of Pharmacoepidemiology and Pharmacoeconomics, Brigham and Women’s Hospital and Harvard Medical School, Boston, MA 02115, USA; Department of Neurology, Massachusetts General Hospital and Harvard Medical School, Boston, MA 02114, USA; Department of Neurology, Massachusetts General Hospital and Harvard Medical School, Boston, MA 02114, USA; Department of Neurology, Jose Reyes Memorial Medical Center, Manila, Metro Manila, 1012Philippines; Department of Neurology, Massachusetts General Hospital and Harvard Medical School, Boston, MA 02114, USA; Sunshine Care Foundation, The Health Centrum, Roxas City, Capiz 5800Philippines; Sunshine Care Foundation, The Health Centrum, Roxas City, Capiz 5800Philippines; Sunshine Care Foundation, The Health Centrum, Roxas City, Capiz 5800Philippines; Department of Neurology, Massachusetts General Hospital and Harvard Medical School, Boston, MA 02114, USA; The Collaborative Center for X-linked Dystonia-Parkinsonism, Massachusetts General Hospital, Charlestown, MA 02129, USA; Department of Neurology, Massachusetts General Hospital and Harvard Medical School, Boston, MA 02114, USA; The Collaborative Center for X-linked Dystonia-Parkinsonism, Massachusetts General Hospital, Charlestown, MA 02129, USA; Department of Neurology, Massachusetts General Hospital and Harvard Medical School, Boston, MA 02114, USA; Department of Neurology, Massachusetts General Hospital and Harvard Medical School, Boston, MA 02114, USA; The Collaborative Center for X-linked Dystonia-Parkinsonism, Massachusetts General Hospital, Charlestown, MA 02129, USA; Department of Neurology, Massachusetts General Hospital and Harvard Medical School, Boston, MA 02114, USA; The Collaborative Center for X-linked Dystonia-Parkinsonism, Massachusetts General Hospital, Charlestown, MA 02129, USA; Department of Communication Sciences and Disorders, MGH Institute of Health Professions, Charlestown, MA 02129, USA; Department of Communication Sciences and Disorders, MGH Institute of Health Professions, Charlestown, MA 02129, USA; Department of Biostatistics, Harvard T.H. Chan School of Public Health, Boston, MA 02115, USA; Department of Neurology, Massachusetts General Hospital and Harvard Medical School, Boston, MA 02114, USA; The Collaborative Center for X-linked Dystonia-Parkinsonism, Massachusetts General Hospital, Charlestown, MA 02129, USA

**Keywords:** natural history, X-linked, dystonia, parkinsonism

## Abstract

X-linked dystonia parkinsonism is a neurodegenerative movement disorder that affects men whose mothers originate from the island of Panay, Philippines. Current evidence indicates that the most likely cause is an expansion in the *TAF1* gene that may be amenable to treatment. To prepare for clinical trials of therapeutic candidates for X-linked dystonia parkinsonism, we focused on the identification of quantitative phenotypic measures that are most strongly associated with disease progression. Our main objective is to establish a comprehensive, quantitative assessment of movement dysfunction and bulbar motor impairments that are sensitive and specific to disease progression in persons with X-linked dystonia parkinsonism. These measures will set the stage for future treatment trials. We enrolled patients with X-linked dystonia parkinsonism and performed a comprehensive oromotor, speech and neurological assessment. Measurements included patient-reported questionnaires regarding daily living activities and both neurologist-rated movement scales and objective quantitative measures of bulbar function and nutritional status. Patients were followed for 18 months from the date of enrollment and evaluated every 6 months during that period. We analysed a total of 87 men: 29 were gene-positive and had symptoms at enrollment, seven were gene-positive and had no symptoms at enrollment and 51 were gene-negative. We identified measures that displayed a significant change over the study. We used principal variables analysis to identify a minimal battery of 21 measures that explains 67.3% of the variance over the course of the study. These measures included patient-reported, clinician-rated and objective quantitative outcomes that may serve as endpoints in future clinical trials.

## Teaser

Acuna *et al.* analysed a total of 87 men: 36 were gene-positive and 51 were gene-negative. We identified a minimal battery of 21 measures that explains 67.3% of the variance over the course of the study and these may serve as endpoints in future clinical trials.

## Graphical Abstract

**Graphical Abstract:**
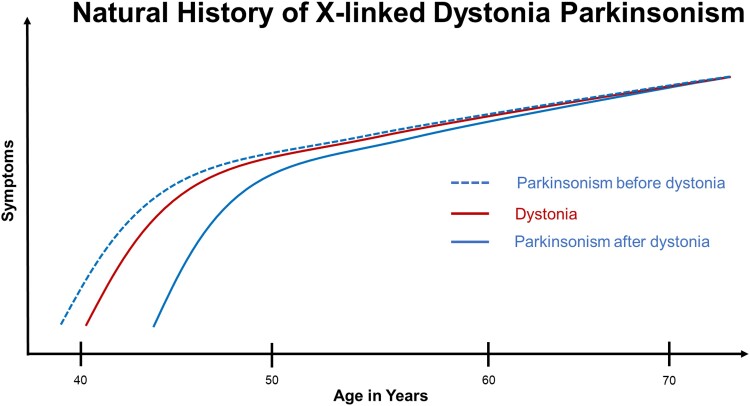

See Haq and Brashear (https://doi.org/10.1093/braincomms/fcad125) for a scientific commentary on this article.

## Introduction

Rare neurodegenerative diseases can offer both powerful opportunities and significant challenges for biomedical research. Deciphering the pathogenic mechanisms underlying such syndromes may offer insight into more common disorders that share phenotypic features, while potentially enabling discovery of therapeutic targets with broad applicability. Yet because they are rare, it can be difficult to assemble patient cohorts that are large enough to achieve statistical power in clinical studies. These difficulties can be further compounded in rare diseases affecting isolated populations in resource-limited geographic regions. X-linked dystonia parkinsonism (XDP, *DYT/PARK-TAF1*) is a neurodegenerative disease presenting all these challenges. It is a movement disorder involving a combination of parkinsonism and dystonia that affects men whose maternal ancestry can be traced to the island of Panay, Philippines.^1^ Genomic studies have revealed that XDP is associated with a disease-specific insertion of a SINE-VNTR-Alu (SVA)-type retrotransposon within an intron of the *TAF1* gene.^2,3^ The SVA contains a hexameric sequence, (CCCTCT)_n_, that is expanded to variable extents among XDP patients, and its length is inversely correlated to age of onset.^4^ In XDP cell models, the SVA induces aberrant mRNA splicing and partial intron retention that decreases levels of the full-length *TAF1* transcript, along with downstream disruptions in multiple gene co-expression networks.^3^ These data suggest potential cellular mechanisms and targets that could be opportunities for drug development in XDP, highlighting the importance of establishing clinical platforms that could support future trials to test candidate interventions.

A frequent issue in designing clinical trial platforms for rare disorders is that the small numbers of available patients may be insufficient to perform traditional randomized studies involving comparisons to placebo or currently available treatment. Given this challenge, regulatory agencies have increasingly accepted comparisons to non-concurrent external controls, the most common of which is prior natural history data.^5-7^ Natural history studies are critical for laying the groundwork for clinical trial infrastructure particularly by identifying quantitative outcome measures that could serve as trial endpoints. Although previous studies of XDP have provided initial descriptions of clinical disease manifestation in patient cohorts, to date, there is little information about measures that could be used to reliably quantify disease progression over time and/or assess the response to an intervention.

A general challenge is that XDP is a combined movement disorder with a unique and diverse phenomenology and considerable clinical heterogeneity.^4,8-11^ Most patients described in the literature exhibited dystonia in the early stages that shifted over time to the hypokinetic features typical of parkinsonism.^12^ However, other studies have documented XDP patients that exhibited parkinsonism as the initial manifestation without overt dystonia.^13^ A consensus among these reports is that XDP symptoms typically emerge at an average age of 39.7 years, although a relatively wide age range for disease onset has been reported.^14,15^ These data suggest that the full phenotypic spectrum of XDP has not yet been captured and that different populations may exhibit different manifestations as well as disease trajectories. Moreover, some of the particularly bothersome symptoms in many XDP patients have not been fully characterized, including (i) impaired speech (a complex combination of hypophonia, hypokinetic/hyperkinetic dysarthria and laryngeal dystonia); and (ii) dysphagia, which can become profound and result in a risk of aspiration.^15^

Here we performed a pilot natural history study to further define disease progression in XDP and advance clinical trial readiness. Towards that objective, we established a longitudinal XDP patient cohort in a predominantly rural region of Panay with the highest reported density of cases and identified measures that could be obtained in remote locations without the need for continuous internet access or reliable electricity. In addition, we developed novel computational tools for assessing relationships among the measures and distinguishing different patterns of disease progression within a relatively small cohort of individuals. The results established comprehensive, quantitative measurements of movement dysfunction and bulbar motor impairments in XDP that may be sensitive and specific to disease progression for use as potential endpoints for future clinical trials.

## Materials and methods

The Institutional Review Board (IRB) approval was obtained from the Philippines IRB at Jose Reyes Hospital, Manila, Philippines. All participants gave written informed consent according to the Declaration of Helsinki, and de-identified data were shared with the Dystonia Partners Research Bank, and analysed in accordance with the Mass General Brigham IRB in Boston, USA. Consent included the explicit, written understanding that no genetic results would be returned.

### Participants

Participants were recruited from the Sunshine Care Foundation clinic in Roxas City, Philippines, and the Jose Reyes Medical Memorial Center in Manila, Philippines. Inclusion criteria were men aged ≥18 years, displaying symptoms of dystonia and/or parkinsonism, confirmed to have the *TAF1* SVA repeat expansion on research testing. In addition, male relatives of participants satisfying these criteria were included.

### Data collection

#### Neurological assessment

We performed a prospective, longitudinal natural history study. All participants underwent a standardized examination at 6-month intervals (±4 weeks) over an 18-month period between September 2017 and October 2019. At each visit, a standardized videotape examination was performed by a trained clinical research coordinator (Supplementary material) that allowed for subsequent quantification of both parkinsonism with the Movement Disorders Society Unified Parkinson’s Disease Rating Scale (MDS-UPDRS),^16^ and dystonia with the Burke–Fahn–Marsden Dystonia Rating Scale (BFM).^17^ During the in-person visit, a trained movement disorders neurologist (C.G., M.S.W. or J.d.G.) conducted the MDS-UPDRS Part 3 rigidity test. Subsequently, the remainder of the MDS-UPDRS Part 3 and BFM Movement scale was performed through simultaneous review of the standardized video tape examination by a team of four movement disorder specialists (J.d.G., M.S.W., A.H. and N.S.s), who were blinded to genotype, to ensure that there was consensus on the severity of symptoms. In addition, during the in-person visit, a standardized history was obtained by trained genetic counselors, height and weight were obtained and speech and oromotor measures were acquired (Table 1).

**Table 1:** Summary of measures that were assessed and the data-value type for each

Group	# of items/measures	Measure type
MDS-UPDRS Part 1	13	5 ordinal categories: 0–4
MDS-UPDRS Part 2	13	5 ordinal categories: 0–4
MDS-UPDRS Part 3	33	5 ordinal categories: 0–4
BFM disability scale	6	5 ordinal categories: 0–4
BFM disability scale: walking	1	6 ordinal categories (0–4, 6)
BFM movement scale	9	10 distinct categories (0,1,2,3,4,6,8,9,12,16)
EAT-10 survey	10	5 ordinal categories: 0–4
CPIB survey	10	4 ordinal categories: 0–3
Lip strength	1	Non-negative integer-valued after Rounding
Tongue strength	1	Positive real-valued
Maximum phonation time	1	Positive real-valued
DDK counts	4	Non-negative integer-valued
DDK durations	4	Positive real-valued
Swallow duration	1	Positive real-valued

#### Patient-reported outcome measures

Five patient-reported outcome measures were translated into the participants’ native language (Hiligaynon (Ilonggo) or Akeanon), and administered verbally by trained research coordinators and community advocates to eliminate the influence of variations in literacy among participants. The MDS-UPDRS Patient Questionnaire (Part 1, non-motor aspects of experiences of daily living; Part 2, motor aspects of experiences of daily living) and the BFM disability Scale were used to assess for motor and non-motor symptoms associated with parkinsonism and dystonia. The Eating Assessment Tool (EAT-10) was used to evaluate participants’ perception of swallowing impairment.^18^ The Communicative Participation Item Bank (CPIB) short form was used to assess the extent to which communication disorders affected participants’ involvement in a wide range of situations that require speech.^19^

#### Oromotor measures

Speech and oromotor measures were performed in the participant’s first language, Filipino, Hiligaynon (Ilonggo) or Akeanon, and analysed by experienced neurologists bi-lingual in Filipino and English (J.d.G., M.S.W.).

Respiratory function for speech was assessed using maximum phonation time (MPT). The MPT was obtained by asking participants to take a deep breath and hold a sustained ‘ah’, as described.^20^ Measures of MPT have shown strong inter-rater reliability (ICC = 0.998).^20^ Oromotor muscles were tested by measuring lip and tongue strength, and maximum syllable repetition rate. Tongue and lip strength were measured in kilopascal (kPA) using the Iowa Oral Performance Instrument (IOPI; IOPI Medical Inc.) as described.^21^ Previous studies have shown reliable measures for tongue strength, particularly after a practice trial.^21^ Maximum syllable rates were tested using two variations of a diadochokinetic (DDK) task. The Alternating Motion Rate (AMR) evaluates the maximum rate of oral muscle contraction by having participants produce single syllables in sequence (/ba/,/da/,/ka/) as rapidly and accurately as possible, for as long as possible on one breath. The Sequential Motion Rate (SMR) places greater demands on articulatory coordination.^22^ Participants were asked to repeat the three-syllable sequence /BaDaKa/ as rapidly and accurately as possible, for as long as possible on one breath. These syllables are within the participants’ native language sound system. Measures of DDK rate show strong inter-rater reliability (ICC = 0.996).^23^ Production of AMR and SMR tasks was recorded to a digital video recorder using high-resolution audio setting (i.e. 44k, 16 bit) and analysed offline, as described.^20^

#### Swallow duration

Participants were instructed to drink three ounces of water in consecutive sips without stopping.^24^ The total length of time to complete the task was measured in seconds.

## Statistical analysis

Since multiple in-depth statistical analyses were performed, we have divided our description into the following sections below: (i) testing for change over the study period, (ii) correlation among measures, (ii) symptom trajectory model and (iv) rate heterogeneity analysis.

## Testing for change over the study period

We performed statistical hypotheses testing for a change in each measure from enrollment to 18 months. For each measure, we applied a Wilcoxon signed-rank test to determine the differences between the first observed value and the last observed value for each participant. In other words, if the measure was missing at 18 months, then we imputed it using the last observation carried forward, and if the measure was missing at enrollment, then we imputed it using the next observation carried backward.^25^ For each test, we included only symptomatic gene-positive males (that is, males with a reported age at onset and genetically confirmed XDP) for which at least two timepoints were available for the measure being tested. In the Wilcoxon signed-rank test, zeros (that is, differences equal to zero) and ties (that is, multiple data points with the same difference) were handled by using the reduced sample procedure (that is, excluding zeros) and the average rank procedure, respectively. To adjust for multiple comparisons, we controlled the false discovery rate (FDR) using the Benjamini–Hochberg procedure. We tested the measures listed in Table 1, and participant’s weight. We combined the 10 CPIB measures into a single sum of the overall CPIB measure since they are very strongly correlated.

### Correlations among measures

To understand the dependencies among measures, we employed a multivariate normal Bayesian model to estimate the correlation for each pair of measures for symptomatic gene-positive males; see Supplementary Material for details. This approach enabled us to handle the missing data in a way that yielded a valid correlation matrix, in contrast to the naive approach of computing the pairwise sample correlation of non-missing entries. Approximately 9% of entries were missing; see Supplementary Material for details (Supplementary Fig. 1). For most measures, higher scores tend to indicate greater disease severity, however, some measures are thought to decrease with disease severity, namely the CPIB and BaDaKa measures, lip strength, tongue strength, MPT, height, weight and BMI. When analysing correlations, we multiplied these measures by −1 for consistency. To identify a subset of measures that (i) indicate disease progression over time and (ii) capture as much information as possible across all measures, we used the Wilcoxon *P*-values (described above) along with principal variables analysis based on our estimated correlation matrix.^26^

### Symptom trajectory model

Using data from both gene-positive (*n* = 29 symptomatic and *n* = 7 pre-symptomatic) and gene-negative (*n* = 51) males, we developed a probabilistic model for how each measure progresses over time—both before and after disease onset. This model allows us to estimate the average trends in symptom trajectories, as well as the variation around this average.

A key challenge is that participants are observed for a relatively short length of time and are at different stages of progression. In addition, the participants were already symptomatic upon enrollment, and thus we relied on individual recall regarding age of onset. To align participants on a common temporal scale, we model progression as a function of age minus an estimated age at onset. This approach, sometimes referred to as ‘entry time realignment’,^27^ has been employed to analyse disease progression for a variety of long-term natural history studies, including Alzheimer’s disease,^28^ exudative age-related macular degeneration,^29^ autosomal recessive Stargardt disease,^30^ Parkinson’s disease^31^ and Huntington disease.^32^ At first, it might seem more natural to model progression as a function of age. However, this is problematic since some older subjects are early in their progression, and some younger subjects are later in their progression; thus, age itself has relatively little to do with disease progression. In contrast, empirically we find that symptom severity is very clearly linked to age minus onset.

Fitting our model involves inferring this estimated age at onset for each symptomatic gene-positive participant and marking the time at which gene-positive symptom trajectories begin departing from gene-negative participants. The estimated age at onset is based on the participant’s reported age at onset, their SVA repeat size and all their observed symptom measures. The main advantage of using an estimated age at onset rather than the reported age at onset is that it makes the results robust to misreporting error, since we do not rely completely on the participants’ memory. Note that entry time realignment has been successfully applied to studies where only symptom measures are available.^27^ The model also involves estimating a rate of progression for each symptomatic gene-positive participant. The estimated rate of progression depends on a participant’s observed symptom measures and estimated age at onset. To fit this model, we pooled information across the 107 measures in Table 1.

To express our model mathematically, we use the following notation: $Y_{ijt}$ denotes the value of measure *j* for subject *i* at visit *t*, and ${\mathrm{Age}}_{it}$ denotes the age of subject *i* at visit *t*. For each symptomatic gene-positive participant *i*, we use ${\mathrm{Onset}}_{i}$ and ${\mathrm{Rate}}_{i}$ to denote the participant-specific parameters ‘age at onset’ and ‘rate of progression’, respectively. We write $[{\mathrm{Age}}_{it}-{\mathrm{Onset}}_{i}{]}_{+}$ to denote the amount of time since onset for participant *i* at visit *t*; this quantity equals $\mathrm{Ag}e_{it}-\mathrm{Onse}t_{i}$ if $\mathrm{Ag}e_{it}>\mathrm{Onse}t_{i}$, and equals zero otherwise. We use $[\mathrm{Ag}e_{it}-\mathrm{Onse}t_{i}{]}_{+}$ as a covariate since patients appear to show little to no measurable signs of disease until onset; further, by including both Age and $[\mathrm{Ag}e_{it}-\mathrm{Onse}t_{i}{]}_{+}$ as covariates, we can use data from gene-negative and asymptomatic gene-positive patients (in addition to symptomatic gene-positive patients) to estimate the parameters of the model. For each categorical measure, we use a cumulative logit model^33^ such that


$$\begin{array}{rl} \mathrm{logit}(P(Y_{ijt}\in \{c_{1},\ldots ,c_{k}\}))= & \;\alpha_{\;jk}+\beta_{\;j1}\mathrm{Ag}e_{it}\\ & +\beta_{\;j2}\mathrm{Rat}e_{i}[\mathrm{Ag}e_{it}-\mathrm{Onse}t_{i}{]}_{+} \end{array}$$

for k=1,…,K where $c_{1},\ldots ,c_{K}$ are the *K* ordinal values that measurement *j* can take. For each *j*, $\alpha_{\;j1},\ldots ,\alpha_{\;jK}$, $\beta_{\;j1}$ and $\beta_{\;j2}$ are real-valued parameters that we infer using the data. Note that the $\alpha_{\;jk}$’s must satisfy $\alpha_{\;j1}<\alpha_{\;j2}<\cdots <\alpha_{\;jK}$. For the positive real-valued measures, we use a log-normal regression model such that


$$\log\;(Y_{ijt})∼\mathrm{Normal}(\alpha_{j}+\beta_{\;j1}\mathrm{Ag}e_{it}+\beta_{\;j2}\mathrm{Rat}e_{i}{\left[\mathrm{Ag}e_{it}-\mathrm{Onse}t_{i}\right]}_{+},\sigma_{j})$$

where Normal (μ,σ) denotes a normal distribution with mean *μ* and standard deviation *σ*. For each *j*, $\alpha_{j}$, $\beta_{\;j1}$ and $\beta_{\;j2}$ are real-valued parameters, whereas $\sigma_{j}$ is a positive real-valued parameter. For the non-negative integer measures, we use a Poisson regression model with


$$Y_{ijt}∼\mathrm{Poisson}(\lambda_{ijt}\;\log(1+\exp(\alpha_{j}+\beta_{\;j1}\mathrm{Ag}e_{it}+\beta_{\;j2}\mathrm{Rat}e_{i}{\left[\mathrm{Ag}e_{it}-\mathrm{Onse}t_{i}\right]}_{+}))$$

where Poisson(λ) denotes a Poisson distribution with mean λ. For each *j*, $\alpha_{j}$, $\beta_{\;j1}$ and $\beta_{\;j2}$ are real-valued parameters that we infer using the data. For lip strength, the offset $\lambda_{ijt}$ is set to 1, while for the DDK count measures, $\lambda_{ijt}$ is set to the corresponding DDK duration measure.

Missing data are handled by analytically marginalizing out any missing values, which—due to the conditional independence assumptions of the model—is implemented by simply dropping the corresponding factors from the likelihood. While this approach does implicitly assume that data are missing at random, violations of this assumption are likely to have minimal effect since most of the missing entries are due to logistical factors unrelated to disease severity.

We fit this model to the data for all 107 measures (see Table 1) within a hierarchical Bayesian inference framework, implemented in the Stan probabilistic programming package.^34^ For each parameter of interest (namely the ages at onset and rates of progression), we use its posterior mean as a point estimate. These posterior means are computed using Markov chain Monte Carlo as implemented in Stan. Further details are in Supplementary material.

### Rate heterogeneity analysis

A limitation of the symptom trajectory model described above is that each participant has a single global rate for all the disease measures. However, it is likely that some participants progress more rapidly in certain types of symptoms and less rapidly in others. Defining a rate profile for each participant would provide valuable insight into the heterogeneity in progression trajectories exhibited by different participants.

To this end, we used our symptom trajectory model to estimate each symptomatic gene-positive participant’s rate of progression along several categories of disease measures. Each category is a group of measures that is pre-defined according to specialist’s knowledge of disease patterns (Supplementary Fig. 2). For each category, independently, we refitted the symptom trajectory model to the subset of measures in that category, using a common time realignment across all the categories. Specifically, we held the estimated ages at onset fixed at the values inferred based on fitting to all measures jointly. Missing data were handled in the same way as before.

This approach extends the entry time alignment models to the setting where each measure category has a different rate of progression for each gene-positive individual. Similar approaches have been applied to model Alzheimer’s progression.^28,35-37^ We chose this approach due to its interpretability (since each category has a clear meaning) and its simplicity (since it is implemented by just applying our symptom trajectory model to various predefined subsets of measures, without having to search over all possible subsets).

**Figure 1:**
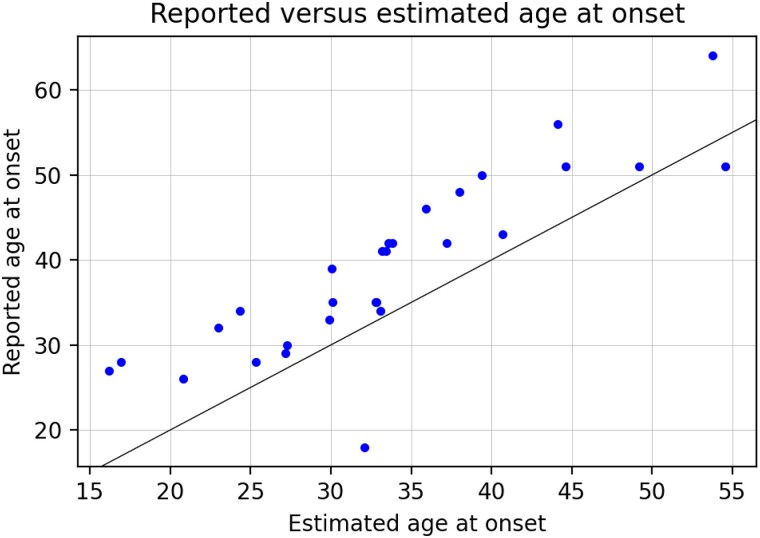
**Age of onset.** Reported age of onset (provided by the subject) versus estimated age of onset (inferred by the model). Data shown for symptomatic gene-positive males (*n* = 29).

This yields an estimated rate of progression $\mathrm{Rat}e_{ig}$ for each gene-positive participant *i* and each category *g*. These rates reveal heterogeneity across individuals and categories, showing whether different individuals progress faster or slower with respect to different categories. Body mass index (BMI) is included as a category in this analysis since it is an important metric of wellness, whereas BMI was excluded from the symptom trajectory model (Table 1) to facilitate validation of the estimated progression summary from that model (Fig. 1). Like the other positive real-valued measures, BMI is modelled using log-normal regression.

### Data availability

Standardized DNA samples (and phenotype data) were obtained from the Dystonia Partners Research Bank. De-identified data that support the findings are available upon reasonable request.

## Results

### Participants

Demographic features and study measurements in SVA-positive men are shown in Tables 2–4.

**Table 2:** Demographics of SVA positive men

	Total (*n* = 36)	Symptomatic men (*n* = 29)	Asymptomatic men (*n* = 7)	*Z* statistic	*P*-value
Age at onset, median (IQR)	39 (32–46)	39 (32–46)	N/A		
Age at exam, median (IQR)	38 (30–48.5)	43 (34–51)	29 (25–34)	−3.04	0.002
Disease duration at enrollment (years) median, (IQR)	4 (2–5)	4 (2–5)	N/A		
TAF1 repeat size	42.5 (40.5–47)	42 (39–47)	43 (42–50)	1.11	0.27
Initial symptom					
Dystonia (*n*)	23	23	N/A		
Parkinsonism (*n*)	6	6	N/A		

The average age of all gene-positive men, at enrollment, was 39.9 years (+11.01, *n* = 36) compared to 36.18 years in the gene-negative cohort (+12.56, *n* = 51). IQR: interquartile range, N/A: Not Applicable.

**Table 3:** Patient-reported motor and non-motor scores of SVA positive men

	Symptomatic men (*n* = 29)				Asymptomatic men (*n* = 7)			
Variable	Baseline	Month 6	Month 12	Month 18	Baseline	Month 6	Month 12	Month 18
Patient-reported								
MDS-UPDRS Part I—complex behaviours								
Mean (SD)	2.54 (2.25)	2.67 (2.57)	2.83 (2.17)	2.92 (2.60)	0.14 (0.38)	0.67 (1.21)	0.2 (0.45)	0
Min–Max	0–10	0–10	0–8	0–9	0–1	0–3	0–1	0–0
Missing	1	2	6	4	0	1	3	5
MDS-UPDRS Part I—non-motor aspects of daily living								
Mean (SD)	3.86 (4.12)	4.15 (3.63)	3.26 (3.24)	5 (3.33)	0.71 (1.11)	0.5 (0.55)	0.2 (0.45)	1 (1.41)
Min–Max	0–13	0–14	0–12	0–12	0–3	0–1	0–1	0–2
Missing	1	2	6	4	0	1	3	5
MDS-UPDRS Part II—motor aspects of daily living								
Mean (SD)	16.68 (12.88)	17.67 (12.05)	20.26 (9.52)	21.72 (12.31)	0.57 (1.13)	0.17 (0.41)	0	0.5 (0.71)
Min–Max	0–48	1–47	1–33	1–44	0–3	0–1	0	0–1
Missing	1	2	6	4	0	1	3	5
BFM disability								
Mean (SD)	8.36 (6.52)	10.51 (6.43)	12.56 (6.60)	13.68 (7.48)	0 (0)	0(0)	0 (0)	0 (0)
Min–Max	0–28	0–26	0–25	0–26	0–0	0	0	0
Missing	1	2	6	4	0	1	3	5

SD—standard deviation.

**Table 4:** Clinician-rated motor scores of SVA positive men

	Symptomatic men (*n* = 29)				Asymptomatic men (*n* = 7)			
Variable	Baseline	Month 6	Month 12	Month 18	Baseline	Month 6	Month 12	Month 18
Clinician-rated								
Nutritional state								
BMI								
Mean (SD)	21.53 (3.69)	20.00 (3.56)	19.12 (3.36)	18.52 (3.07)	23.31 (5.05)	24.56 (3.98)	21.59 (4.44)	22.67 (3.24)
Min–Max	16.84–26.26	14.47–27.07	13.87–26.67	14.09–25.68	17.45–29.26	18.19–27.95	16.82–25.71	20.34–24.96
Missing	22	2	6	4	3	1	3	5
Swallow mechanism								
Lip strength								
Mean (SD)	13.31 (6.79)	13.01 (5.46)	11.13 (4.84)	16.59 (7.57)	27.33 (7.36)	24 (7.27)	32.17 (0.71)	32 (10.84)
Min–Max	1.67–30	2–21.3	3.67–17.67	4.33–33.67	20.33–35	15.67–32	31.67–32.67	24.33–39.67
Missing	5	5	13	8	4	2	5	5
Tongue strength								
Mean (SD)	32.34 (17.77)	33.58 (14.79)	30.96 (16.09)	30.55 (15.19)	61.33 (7.23)	51.73 (8.21)	67.34 (4.72)	53 (19.80)
Min–Max	4.67–65	10.67–61.67	9.33–57.33	8–57.67	53–66	42.33–64.67	64–70.67	39–67
Missing	5	3	12	7	4	2	5	5
Swallow duration								
Mean (SD)	8.87 (7.56)	7.25 (4.84)	10.51 (6.66)	12.06 (9.07)	2.33 (0.32)	2.86 (0.63)	2.22 (0.33)	2.97 (0.65)
Min–Max	0.7–31	2.3–25.03	2.83–28	3.1–37.7	2–2.64	2.38–3.89	1.98–2.45	2.51–3.43
Missing	5	5	15	10	4	2	5	5
Communication ability								
SMR BA rate								
Mean (SD)	5.21 (1.17)	5.17 (1.07)	5.05 (1.19)	4.56 (1.29)	6.02 (0.32)	6.28 (0.45)	4.41 (1.2)	4.01
Min–Max	2.7–7.4	2.8–7.2	2.98–6.97	2.37–6.65	5.72–6.35	5.8–7.0	3.43–6.4	4.01
Missing	14	10	12	18	4	2	2	6
SMR DA rate								
Mean (SD)	5.11 (1.66)	4.73 (1.28)	4.57 (1.55)	3.94 (1.79)	6.57 (0.28)	6.56 (0.55)	5.4 (1.82)	3.9
Min–Max	2.1–8.1	2.4–6.5	2.23–6.84	1.23–6.11	6.3–6.85	6–7.4	3.76–7.65	3.9
Missing	15	9	12	18	6	4	4	8
SMR KA rate								
Mean (SD)	4.74 (1.20)	4.93 (1.61)	5.03 (2.02)	4.07 (1.63)	5.83 (0.51)	5.9 (0.57)	4.98 (1.55)	3.64
Min–Max	2.6–6.9	2.6–8.8	1.98–8.83	1.37–6.42	5.25–6.2	5.4–6.8	3.25–6.78	3.64
Missing	16	13	12	18	6	4	4	8
AMR rate								
Mean (SD)	1.98 (0.40)	1.71 (0.32)	1.82 (0.56)	1.70 (0.54)	2.41 (0.45)	2.22 (0.15)	2.47 (0.16)	1.92
Min–Max	1.25–2.6	1.1–2.4	0.75–2.78	0.64–2.41	1.9–2.75	2–2.4	2.25–2.66	1.92
Missing	14	9	13	18	4	2	2	6
MDS-UPDRS Part III								
Mean (SD)	39.86 (18.21)	35.38 (16.57)	36.27 (11.84)	51.04 (20.73)	8 (9.26)	3.6 (5.37)	2.25 (2.63)	2.5 (0.71)
Min–Max	10–90	3–80	8–53	0–92	2–27	0–13	0–6	2–3
Missing	0	3	6	4	0	2	3	5
BFM movement								
Mean (SD)	24.21 (23.96)	27.83 (26.00)	28.68 (23.61)	43.96 (28.31)	0.57 (1.51)	0	0.25 (0.5)	0
Min–Max	0–96	0–91	1.5–88	3–92	0–4	0	0–1	0
Missing	0	3	7	4	0	2	3	5

SD—standard deviation

### Testing for change over the study period

Of the 99 measures tested (Table 1, with CPIB combined into one measure, and participant weight), we found that nine measures exhibited a significant change, controlling FDR at 0.05 (Table 5). This demonstrates that certain measures exhibit a significant change over time; however, it can be refined by (i) accounting for subject heterogeneity, for instance, in terms of age and disease progression, and (i) using data from all timepoints. Results for each measure are shown in Supplementary Fig. 3.

**Table 5:** Measures exhibiting significant change over 18 months adjusted for multiple testing

Measure	*n*	mean	stder*r*	W	*P*-value	BH *P*-adjusted
Weight	26	−8.87	2.24	−261	0.0002	0.016
BFM disability: feeding	27	1.07	0.24	91	0.0002	0.012
BFM disability: dressing	27	0.93	0.22	91	0.0002	0.008
UPDRS 3.2: facial expression	28	0.64	0.16	78	0.0005	0.012
BFM disability: speech	27	0.78	0.21	96	0.0011	0.022
BFM disability: hygiene	27	0.85	0.23	96	0.0011	0.018
UPDRS 3.11: freezing of gait	28	1.14	0.30	142	0.0019	0.027
EAT-10: Q8—food sticks in throat	27	1.00	0.31	90	0.0022	0.027
UPDRS 3.1: speech	27	0.89	0.25	90	0.0035	0.038

For each measure, all participants who had at least two values taken over the course of the study were included. Stderr: standard error, BH *P*-adjusted: Benjamini–Hochberg *P*-value.

## Correlations among measures

We analysed the measures to determine inter-item correlations. Our goal was to determine the smallest number of measures required to effectively monitor all aspects of disease progression, thus reducing the effort required by both participants and study staff in a future clinical trial.

Supplementary Fig. 4 shows a heatmap of the matrix of estimated pairwise correlations among 102 measures, consisting of the 99 measures on which testing for change was performed, along with SVA repeat size, reported age at onset and height. This matrix identifies many strongly correlated pairs of measures, which can be organized into natural clusters with clear interpretations using hierarchical clustering (Supplementary Fig. 5).

We used principal variables analysis to select a minimal battery that contains representatives from as many clusters as possible, preferring measures that change significantly over time and explain as much variance as possible. We proposed using the measures in Table 6 as a minimal battery. Table 6 shows the per cent of total variance explained by each member alone and jointly. Since some measures are naturally taken in pairs (such as BFM movement: right arm and BFM Movement: left arm), we combine these into groups that were selected as a block, resulting in 15 groups, containing 21 measures total. Supplementary Fig. 6 shows the per cent of the variance of each measure that was explained by the minimal battery, and subsequently the amount explained by each member of the minimal battery. Together, the measures in the minimal battery explained 61.4% of the total variance across all measures. When adding reported age at onset, the resulting set of 22 measures explained 67.3% of the total variance.

**Table 6:** Proposed minimal battery of measures that quantify disease progression

Measure	PVE alone	PVE jointly
Weight	10.3%	3.0%
BFM disability: speech	9.9%	3.2%
BFM disability: feeding	18.2%	4.5%
BFM disability: dressing	16.0%	4.8%
BFM movement: arms (right arm and left arm)	14.9%	5.5%
BFM movement: legs (right leg and left leg)	12.8%	4.9%
UPDRS 3.2: facial expression	12.1%	3.5%
UPDRS 3.3: rigidity—upper extremities (RUE and LUE)	8.2%	4.7%
UPDRS 3.7: toe tapping (right foot and left foot)	12.9%	5.5%
UPDRS 3.11: freezing of gait	9.0%	3.0%
UPDRS 3.15: postural tremor of the hands (right and left)	9.9%	4.6%
UPDRS 3.18: constancy of rest tremor	8.1%	4.4%
EAT-10: Q1—my swallowing problem has caused me to lose weight.	17.1%	4.8%
EAT-10: Q8—when I swallow food sticks in my throat.	9.1%	2.7%
DDK: Ka (Ka rate and Ka secs)	8.7%	6.5%

In analysing bradykinesia, there were correlations between left limb and right limb with respect to leg agility, toe tapping, hand movements and pronation/supination (Table 7). Similarly, there was a correlation between the presence of rigidity in the right and left arms, and between the right and left legs. The presence of dystonia in a limb also correlated with dystonia in the contralateral limb.

**Table 7:** Correlations between left and right analogues, where *x* ± *w* denotes a 95% credible interval

Measures	Correlation
UPDRS 3.5: hand movements—Right hand versus Left hand	0.81 ± 0.07
UPDRS 3.6: Pronation-supination—Right hand versus Left hand	0.83 ± 0.07
UPDRS 3.7: toe tapping—right foot versus left foot	0.87 ± 0.06
UPDRS 3.8: leg agility—right leg versus left leg	0.94 ± 0.03
UPDRS 3.3: rigidity—RUE versus LUE	0.74 ± 0.09
UPDRS 3.3: rigidity—RLE versus LLE	0.80 ± 0.07
BFM movement: right arm versus left arm	0.83 ± 0.06
BFM movement: right leg versus left leg	0.84 ± 0.06

We explored correlations between dystonia and bradykinesia in each limb (Table 8). We found that dystonia correlated significantly with bradykinesia assessed by finger/toe tapping and leg agility/hand movements, but not rigidity or rest tremor; here, ‘significance’ was assessed by a 95% interval excluding zero.

**Table 8:** Correlations between corresponding BFM and UPDRS measures

BFM measure	UPDRS measure	Correlation
BFM movement: right leg	UPDRS 3.7: toe tapping—right foot	0.43 ± 0.16
”	UPDRS 3.8: leg agility—right leg	0.34 ± 0.17
”	UPDRS 3.3: rigidity—RLE	0.13 ± 0.19
BFM movement: left leg	UPDRS 3.7: toe tapping—left foot	0.45 ± 0.15
”	UPDRS 3.8: leg agility—left leg	0.35 ± 0.18
”	UPDRS 3.3: rigidity—LLE	0.10 ± 0.19
BFM movement: right arm	UPDRS 3.4: finger tapping—right hand	0.38 ± 0.17
”	UPDRS 3.5: hand movements—right hand	0.32 ± 0.19
”	UPDRS 3.6: pronation-supination—right hand	0.33 ± 0.19
”	UPDRS 3.3: rigidity—RUE	0.07 ± 0.19
”	UPDRS 3.17: rest tremor amplitude—RUE	0.08 ± 0.21
BFM movement: left arm	UPDRS 3.4: Finger tapping—left hand	0.36 ± 0.18
”	UPDRS 3.5: hand movements—left hand	0.35 ± 0.18
”	UPDRS 3.6: pronation-supination—left hand	0.36 ± 0.18
”	UPDRS 3.3: rigidity—LUE	0.10 ± 0.19
”	UPDRS 3.17: rest tremor amplitude—LUE	−0.25 ± 0.26

UPDRS 3.3: rigidity—RLE, UPDRS 3.3: rigidity—LLE, UPDRS 3.3: rigidity—RUE, UPDRS 3.17: rest tremor amplitude—RUE, UPDRS 3.3: rigidity—LUE and UPDRS 3.17: rest tremor amplitude—LUE lack significance as the 95% interval includes zero.

## Associations with repeat size

Age of onset was strongly associated with SVA repeat size (Supplementary Fig. 7, *r* = −0.70, *P* = 2.73e-5). We analysed disease severity for association with SVA repeat size. Since disease severity depends strongly on when a participant is observed over the course of the disease, we tested for association when adjusting for age at exam minus age at onset, in addition to testing without such adjustment. In both analyses, we found no evidence of association of SVA repeat size with any other clinical measure. See Supplementary Fig. 8 for scatterplots and tests of association between SVA repeat size and UPDRS Part 3 total, BFM movement total and BFM disability total.

## Symptom trajectory model

We used our symptom trajectory model to better characterize overall XDP symptom progression and place each subject along this trajectory. In Supplementary Fig. 9, we illustrated how this model provides an estimate of the lifetime trajectory of any given measure for each participant, stitching together data from across all participants to extrapolate beyond the small window of time in which each participant is observed.

Figure 1 shows reported age at onset versus estimated age at onset for symptomatic gene-positive male participants. This analysis suggested that the reported age at onset tended to lag behind the true age at onset by roughly 5–10 years, which may reflect the time required for symptoms to become noticeable.

**Figure 2:**
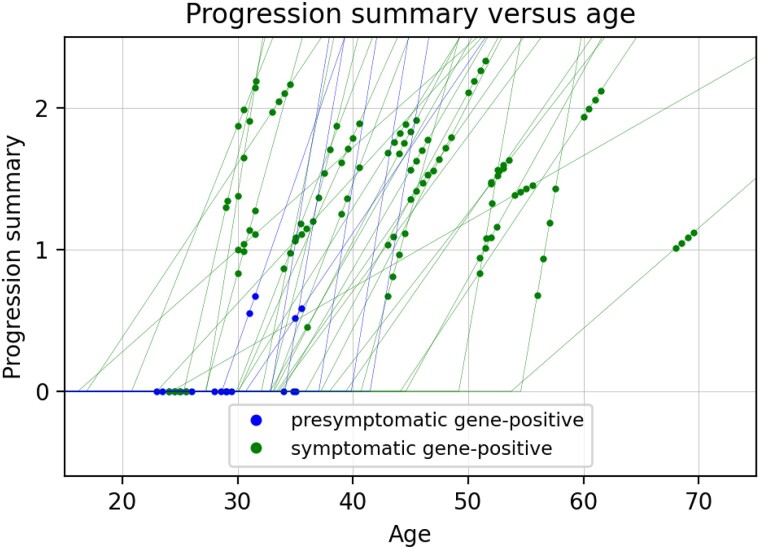
**Model-based summary of progression versus age.** The points show the progression summary value at each visit for each participant, and the thin lines show the estimated piecewise linear trajectory for each participant as a function of age. Data shown for all gene-positive males (*n* = 29 symptomatic, *n* = 7 pre-symptomatic).

The model also involved estimating a rate of progression for each gene-positive subject, which represented the relative rate at which their symptoms were progressing. Combining the estimated rate of progression with the estimated age at onset, we defined a quantitative summary of disease progression for each subject at each point in time. As shown in Fig. 2, this progression summary was defined as equal to zero until reaching the estimated age of onset, and then increases linearly at the estimated rate of progression.

**Figure 3:**
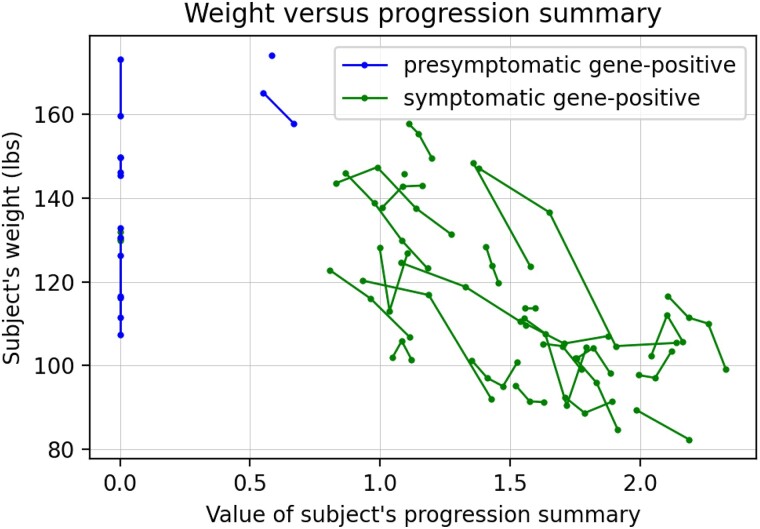
**Participant weight versus the progression summary.** Successive timepoints for the same participant are connected by lines. Data shown for gene-positive males (*n* = 29 symptomatic, *n* = 7 pre-symptomatic). For A, B, C, these are estimates based on the symptom trajectory model; no statistical testing involved.

To assess whether our progression summary provided a useful indicator of disease severity, we considered its association with participant weight, a key metric of wellness. We found that our progression summary was strongly inversely correlated with subject weight (Fig. 3, *r* = −0.6, *P* < 0.00001). As neither weight nor BMI were inputs to this model, this suggests that our progression summary provides a highly relevant metric of disease severity.

As the rate of progression is of central interest for disease prognosis and treatment, it would be useful to predict the rate of progression based on early indicators such as initial symptoms or genetic factors. To explore this possibility, we looked for associations between the estimated rate of progression and SVA repeat size, initial disease symptoms and family ID (Supplementary Fig. 10). Participants who were siblings or first cousins were given the same family ID, as a proxy for the presence of a more similar genetic background that may influence disease progression. We found no clear association between the rate of progression and each of these factors.

## Rate heterogeneity analysis

**Figure 4:**
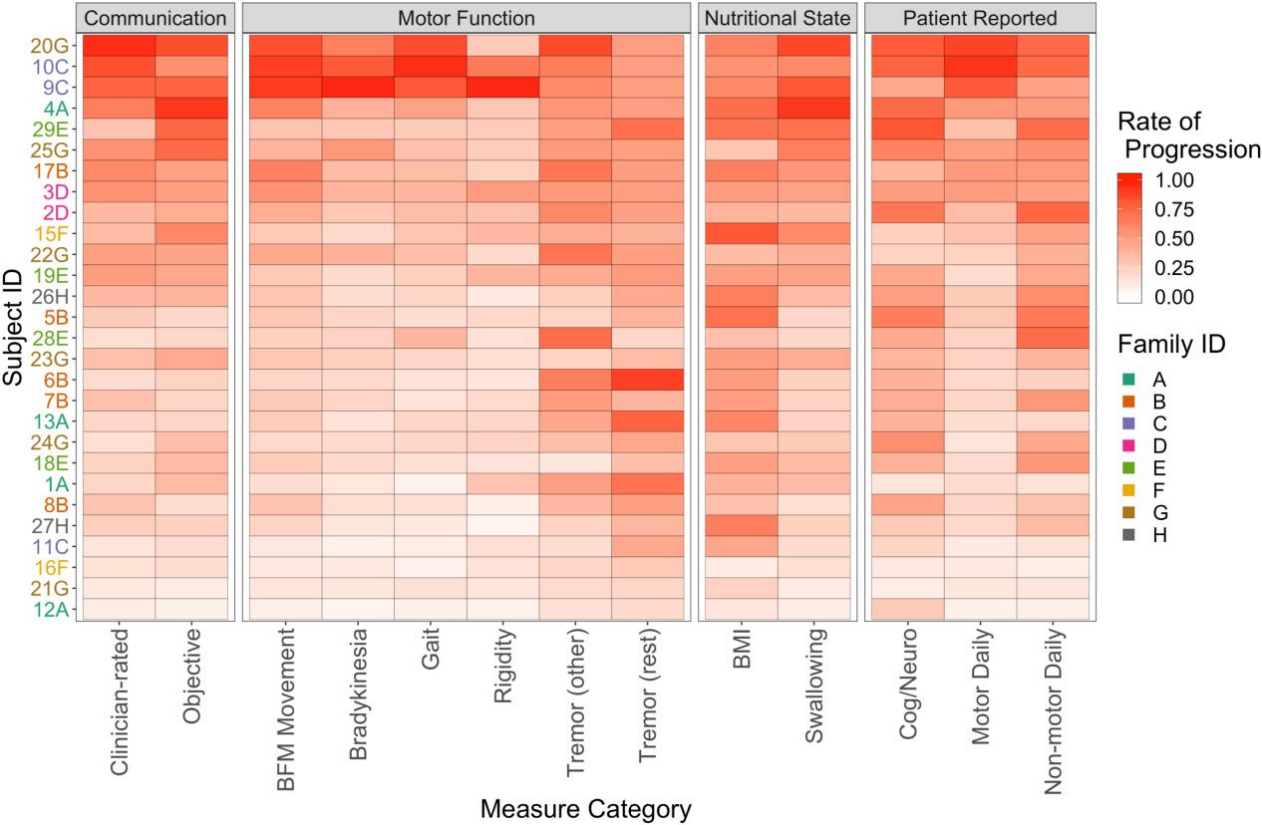
**Estimated rates of progression.** Estimated rates of progression for each pre-defined category, for each symptomatic gene-positive subject, except for subject 14D. Subjects are ordered (highest to lowest) according to their average rate across the 13 categories. Estimates based on the rate heterogeneity analysis; no statistical testing involved.

To further explore disease heterogeneity, we considered 13 pre-defined categories of measures (Supplementary Fig. 2) and used our model to estimate the rate at which each subject is progressing with respect to each category (Fig. 4). This extended the symptom trajectory model by having a vector of rates for each participant (rather than a single global rate), which could be used to characterize different patterns of progression that may occur in different participants. Characterizing different patterns of disease progression may be helpful in developing disease-course specific treatment. We used hierarchical clustering to organize the subjects according to their estimated rates of progression along the 13 pre-defined categories (Supplementary Fig. 11).

There was strong heterogeneity, with some participants progressing rapidly in most categories, while others progressed slowly across all categories. Some categories appeared to exhibit little correlation with most of the other categories, such as tremor (other), tremor (rest) and BMI. As an outlier, participant 6B appeared to be progressing rapidly in only the categories of tremor (rest) and tremor (other).

The Euclidean distance between the estimated rates of progression for each pair of participants (that is, every subset of two subjects) with respect to the 13 categories of disease measure is shown in Supplementary Fig. 12. Three participants (9C, 10C and 20G) stood out as having substantially different and more rapid patterns of estimated progression than the other participants. Further, participants 9C and 10C exhibited especially high rates of progression in the rigidity category. Notably, 9C and 10C were brothers with the same repeat size of 38 (which is on the low end) and the same reported age at onset of 51 (which is on the high end). Despite their relatively late reported age at onset, 9C and 10C exhibited rapid progression over the 18-month study period. Progression in participant 10C was so severe that our model estimated that their onset occurred earlier (age 49) than reported (age 51).

One participant (14D) was not shown in Fig. 4 or Supplementary Fig. 12 as, according to the model estimates, he had not yet reached his age of onset at the time of the study (even though he had reported onset), and as a result, the estimated rates for this subject simply reverted to the prior values, making them uninformative.

## Discussion

Our goal was to identify quantifiable measures of disease progression in XDP that could be performed in a relatively resource-poor setting, for potential use in future clinical trials. We identified nine measures that exhibited a statistically significant change over 18 months in men with symptomatic XDP (controlling FDR at 0.05). These measures included patient-reported (feeding, dressing, speech and hygiene from the BFM disability scale and Question 8 from EAT-10), clinician-reported (UPDRS 3.2: facial expression and UPDRS 3.1: Speech) and objective (weight/BMI) measures. While clinician-rated facial expression may represent a combination of jaw opening dystonia and parkinsonism-related lip parting, its significant decline over 18 months indicated its utility as an end point in a clinical trial. Thus, clinician-rated measures as well as participant’s weight and patient reports of difficulty with dressing, feeding, swallowing and speaking may serve as robust measures of meaningful efficacy in a therapeutic trial.

We assessed for correlations among the chosen measures. We found many strongly correlated pairs of measures, which could be organized into natural clusters with clear interpretations using hierarchical clustering (Supplementary Fig. 5). Notably, the presence of bradykinesia in one limb correlated significantly with its presence in the contralateral limb. This suggests that the parkinsonian features of XDP may occur bilaterally in a more closely associated temporal onset, which is in sharp contrast to the typically unilateral onset with spread over time to become bilateral that is seen in idiopathic Parkinson’s disease (PD). We also found that the presence of dystonia in a limb correlated significantly with the presence of bradykinesia, but not rigidity or tremor, in the same limb. While significant limb dystonia may interfere with the ability to perform repetitive motor tasks, which are the basis for determining the degree of bradykinesia, it is also possible that the onset and progression of dystonia and parkinsonism in XDP are more closely associated and are distinct from that seen in idiopathic PD.

We used principal variables analysis to identify a minimal battery of measures that allowed monitoring of disease progression and explained as much variance as possible. This permitted us to balance the need to minimize strain on participants and study staff while maximizing our ability to fully capture the ways in which disease progression affects different body regions and daily function in various areas, from speech to gait. We identified a minimal battery of 21 measurements, encompassing objective, quantitative measurement of weight and speaking ability (/ka/component of the DDK rate), clinician-rated signs including dystonia in upper and lower limbs, parkinsonism (facial expression, rigidity, bradykinesia, freezing of gait and constancy of rest tremor) and participant-reported difficulties with speaking, feeding oneself and swallowing, which explained 61.4% of the variance seen across all measures, over the 18 months of this natural history study. The /ka/ syllable of the DDK task, which tests posterior tongue movement function, may be more sensitive to neurological differences than the /ba/and/da/ syllables, which test lip and anterior tongue movement, respectively. Similar findings have been reported in patients with amyotrophic lateral sclerosis.^38,39^ These measures may serve as inexpensive, non-invasive and relatively simple tools to assess efficacy in future treatment trials.

Previous studies have found that age at onset is associated with *TAF1* SVA repeat length,^4^ and our data replicate this finding (Supplementary Fig. 7). It has also been reported that measures of disease severity are associated with repeat length, including the BFM and MDS-UPDRS Part 3,^16^ however, we are unable to replicate such associations (Supplementary Fig. 8). While lack of significance does not imply that there is no association, it is possible that the strong associations observed by Westenberger *et al*.^40^are related to confounding, as there was no adjustment for age at examination minus age at onset. We also did not identify an association between rate of disease progression and family ID (Supplementary Fig. 10), which we used as a proxy to group together those participants with a more closely shared genetic background. Further study with a larger sample size is required to assess for predictors of the rate of disease progression more fully.

This study has several limitations. After the onset of our study, an XDP-specific scale was published.^41^ As we had already initiated our study, we were not able to utilize this XDP-specific scale. Our sample size was relatively small, and follow-up beyond 18 months was disrupted due to the COVID pandemic. Tests of cognitive function, particularly executive function, were not included in our initial study. A cross-sectional study of 29 symptomatic XDP participants revealed evidence of cognitive dysfunction, most commonly in attention and executive function, confirming the findings in prior case reports.^42-44^ A recent study of 15 males with the XDP-associated SVA and no physical symptoms displayed no evidence of cognitive dysfunction on standardized screening tests.^45^ Thus, longitudinal tests of cognitive function, adapted to the local language and literacy level of participants, should be a part of a future natural history study. Another limitation was that patients whose symptoms were severe had difficulty performing the speech and swallow measures and were unable to complete that portion of the study. In addition, the use of quantitative motion sensors, which have been used in cross-sectional studies of XDP participants, may be more sensitive to change over time than traditional clinical examination-based scales.^45^ We limited our report to symptomatic males, as our priority was to identify quantitative clinical measures that could be used in a future clinical trial. Some female XDP carriers have also been reported to exhibit movement abnormalities.^46^ Future natural history studies should include both women and men from families with XDP, with enrollment beginning prior to onset of obvious signs and symptoms of disease, to better understand the onset of disease and how frequently female carriers are affected.

The XDP is a heterogeneous disorder, with at least two primary phenotypes identified, those who present with dystonia, which is replaced by parkinsonism, and those who present with parkinsonism that progresses.^11^ Different phenotypes may respond differently to a given treatment. In addition, identifying those who are likely to progress more quickly may identify those in whom the risk benefit ratio for more aggressive treatments is worthwhile. Thus, we sought to create models that may help to predict the rate of progression in each patient. We pooled all the measurements obtained during this study, both objective and participant-reported, and developed a quantitative summary of disease progression for each subject. Importantly, this model of disease progression correlated with declining weight, an independent measure of overall health. This model may be useful in estimating disease progression for a given patient, based on their age and overall disease severity at time of evaluation. Further study in larger sample sizes and over a longer interval are needed to better assess the natural history in XDP.

Finally, the methodology developed in this article may be useful in the study of similar diseases in which entry time realignment models have been employed, such as Alzheimer’s disease,^28,47^ exudative age-related macular degeneration,^29^ autosomal recessive Stargardt disease,^30^ Parkinson’s disease^31^ and Huntington disease.^32^

## Supplementary Material

fcad106_Supplementary_Data

## Abbreviations

AMR =: alternating motion rate
ALS =: amyotrophic lateral sclerosis
BFM =: Burke–Fahn–Marsden
BMI =: body mass index
CPIB =: Communicative Participation Item Bank
DDK =: diadochokinetic
DPRB =: Dystonia Partners Research Bank
DYT =: Dystonia
EAT-10 =: Eating Assessment Tool
FDR =: false discovery rate
FWER =: family-wise type I error rate
IOPI =: Iowa Oral Performance Instrument
ICC =: inter-rater reliability
IRB =: Institutional Review Board
kPA =: kilopascal
LOCF =: last observation carried forward
MCMC =: Markov Chain Monte Carlo
MDS-UPDRS =: Movement Disorder Society Unified Parkinson’s Disease Rating Scale
MPT =: maximum phonation time
NOCB =: next observation carried forward
PARK =: parkinsonism
PD =: Parkinson’s disease
PVE =: per cent of total variance explained
SMR =: sequential motion rate
SVA =: SINE-VNTR-Alu
TAF1 =: TATA-box binding protein associated factor 1
UPDRS =: Unified Parkinson’s Disease Rating Scale
XDP =: X-linked dystonia parkinsonism
