# Supplementary material for: Establishing a natural history of X-linked dystonia parkinsonism
Source: Brain Commun. 2023 Apr 4;5(3):fcad106. doi: 10.1093/braincomms/fcad106 (PMC10231801; doi:10.1093/braincomms/fcad106)
Supplement: fcad106_Supplementary_Data [file fcad106_supplementary_data.docx]

# Supplementary Material

Videotaped Neurological Examination Protocol

**Preparation**:

Ask subject to - 1) Roll-up sleeves to elbow & pants to the knee

2) Remove shoes, socks, coats, scarves, hats, glasses, etc.

3) Tie up/back long hair

4) Have men remove shirt (if trunk dystonia)

Film ID info - 1) ID code

2) Date of videotaping

3) Film ID info for 10 seconds

NOTE: Film entire segment with subject sitting directly opposite you, i.e. Head on shot.

**WHOLE BODY**:

- Ask subject to sit in a chair (preferably a chair without arms and 6” from wall) at rest, feet flat on the floor, arms relaxed with hands sitting on each thigh. Film whole body for 10 seconds.
- Zoom into lower body, feet, upper body and face. While zoomed on chest & face ask the subject to state the months of the year starting from December going backwards. Ask the subject if s/he takes Levodopa/Sinemet. If yes, ask time of most-recent dose/pill.

**HEAD**:

(Zoom on chest & face)

Ask subject to:

- Look straight ahead; relax and let your body do what it wants. Close your eyes for about five seconds.
- Look straight ahead and try to hold your head in a neutral position.
- Slowly turn head to the left, to the right, up, straight ahead, and downward.
- Look straight ahead, slowly tilt head to the right, touching ear to shoulder. Repeat to the left
- Demonstrate any sensory tricks

**FACE**:

(Zoom on face & neck)

Ask subject to:

- Open and close your eyes tightly five times.
- Open and close your jaw five times
- Open your mouth, stick out your tongue, move your tongue side to side; close your mouth

**ARMS AND HANDS**:

(Zoom on upper part of body)

Ask subject to:

- Place arms outstretched straight in front of you, with fingers spread out, and palms down. Close your eyes
- Keep your eyes closed. Slowly turn hands over so that your palms face the ceiling.
- Slowly turn palms down again. Open your eyes.
- First with your right hand, turn your palm to the ceiling then the floor as full and as fast as possible 10 times.
- Now the same with your left hand.
- Make a “wing” position: (both elbows straight out to the side, each elbow bent with palms of hands facing the floor, and both hands facing toward each other; close but not touching)
- Close your eyes and hold 10 seconds

**RAPID SUCCESSIVE MOVEMENTS**:

(Continue zoom on chest & head with focus on hands)

Ask subject to:

- First on the right, make big, quick taps with your first finger and thumbs until I say stop.
- Now the same on the left.
- First on the right, fully open and close fists as though flinging water until I say stop.
- Now the same on the left.

Switching to your feet: (CHANGE zoom so that it includes the feet)

- Stomp foot on the floor until I say stop, lifting entire foot off the floor
- First on the right, tap toes until I say stop keeping heel on the floor
- Tap your heel until I say stop, keeping your toes on the floor
- Tap heel-out / toe-back until I say stop, alternating heel and ball of foot
- Now the same on the left.

**FINGER-TO-NOSE**:

(Zoom on chest & head, getting a close-up of the arm, elbow and finger)

*Hold your finger out in front of the subject about an arm’s length away from the subject’s face; move your finger so subject reaches 2x in each position. Make sure that the subjects is extending his/her arm FULLY when reaching to touch your finger*

Ask subject to:

- Use your index finger to touch your nose, then SLOWLY extend your arm to touch your finger
- Repeat 5 times with each hand.

**RIGIDITY**:

(Zoom on the full body)

Ask the subject to:

- Relax as much as possible

*Slowly test the range of motion and tone of neck, R/LUE, R/LLE; Start with the extremities and end with the neck. With each maneuver, if the tone is normal and with full range of motion, ask the subject to open and close the contralateral hand. CALL OUT EACH RATING*

**VOICE**:

(Zoom on upper body and face)

Ask the subject to:

- Comment on/Talk about the weather to get a 1-minute voice sample.
- Ask whether s/he has swallow problems or choking
- Ask subject to clear his/her throat
- Take deep breath and say:
  - “aaahhh” (as in CAT) for as long as you can. Repeat
  - “eeeeee” for as long as you can. Repeat
- Please read the “Rainbow Passage” and the “Bamboo Passage” (attached);
- If XDP cohort,
  - Take a deep breath and count aloud to the beat of the metronome, starting at ‘1’, until you run out of breath
  - Please say Philippines pledge of allegiance (in Tagalog/Filipino)
  - Please read the following sentences one at a time.
    - *Randomly provide one of the 50 Speech Intelligibility Test (SITS) sheets*
  - *Complete diadochokinetic rate task (Oral-DDK*)
    - Take a deep breath. Say “pa” as quickly and accurately as possible on one breath.
    - Repeat with “ta” and “ka” sounds. Then “pataka”

**STANDING**:

(Zoom to capture the full body – head to toe at full height)

Ask subject to:

- Cross your arms over your chest and stand up unassisted; uncross arms
- Face the camera, then make 4 quarter turns, stopping after each turn for video

**PULL TEST**:

(Continue head-to-toe zoom)

*Be prepared to catch that subject if he/she falls. Stand with your back about 2 feet from a wall.*

Ask subject to:

- Stand relaxed, with feet spread slightly apart.

*Stand about 2 feet behind the subject. Place your hands on the subject’s shoulders and gently shake. Then quickly but gently pull back on the subjects shoulders, allowing the subject to catch his/her balance.*

*Repeat 2-3 times.*

**WALKING**:

(Continue head-to-toe zoom)

Ask subject to:

- Walk up and down long hallway, going back and forth three times.

(CHANGE zoom to focus on lower body)

- Walk toward me heel-toe, as if you’re on a tight rope.
- Walk backwards normally for 15 steps.
- Walk forward on your tip toes for 10 steps.
- Walk forward on your heels for 10 steps.

**WRITING**:

(Zoom on the full body but focus down the arm on the upper body, then zoom on hands)

Ask the subject to WRITE:

- ‘Today is a sunny day in southern California’ three times with his/her dominant hand
- A line of connected cursive L’s/loops from one side of the page to the other; first with dominant, then non-dominant hand
- Create a spiral; start small in the middle of the page and get progressively larger; make sure to keep elbow and wrist off table; first with dominant, then non-dominant hand

*Film the subject’s writing sample page for 10 seconds*

**Eating Assessment Tool (EAT-10)**

**Instructions**:

For each question, please mark to what extent are the following scenarios problematic for you, where 0 = No problem and 4 = Severe problem.

# To what extent are the following scenarios problematic for you?

|  | **0 = No**  **problem** | **1** | **2** | **3** | **4 = Severe problem** |
| --- | --- | --- | --- | --- | --- |
| 1. My swallowing problem has caused me to lose weight.  (1) Ang problema ko sa pagtulon ang rason sa akon pagniwang. |  |  |  |  |  |
| 2. My swallowing problem interferes with my ability to go out for meals.  (2) Ang problema ko sa pagtulon naga pugong sa akon magkaon sa restoran/kalan-an. |  |  |  |  |  |
| 3. Swallowing liquid takes extra effort.  (3) Nabudlayan ko mag inom tubig. |  |  |  |  |  |
| 4. Swallowing solids takes extra effort.  (4) Nabudlayan ko magtulon sang matig-a na  pagkaon. |  |  |  |  |  |
| 5. Swallowing pills takes extra effort.  (5) Nabudlayan ko magtulon sang bulong nga tablet. |  |  |  |  |  |
| 6. Swallowing is painful.  (6) Masakit mag tulon. |  |  |  |  |  |
| 7. The pleasure of eating is affected by my swallowing.  (7) Ang problema ko sa pagtulon ang rason nga dulaan ko gana mag kaon. |  |  |  |  |  |
| 8. When I swallow food sticks in my throat.  (8) Kung magtulon ko sang pagkaon gapilit sa  tutunlan. |  |  |  |  |  |
| 9. I cough when I eat.  (9) Naga ubo ako kung magkaon. |  |  |  |  |  |
| 10. Swallowing is stressful.  (10) Ang pagtulon nakakastress. |  |  |  |  |  |

Total Score:

**Correlation estimation using multivariate normal Bayesian model**

Supplementary Figure 1 summarizes the amount of missing data, both by measure and by subject-visit. Overall, 9% of entries were missing. The measures with the most missing entries were the DDK measures, which were missing approximately 40-45% of entries. However, 80% of measures were missing fewer than 10% of entries. Meanwhile, for a handful of visits there were as many as 40-65% of entries missing; however, for 69% of subject-visits, fewer than 10% of entries were missing.


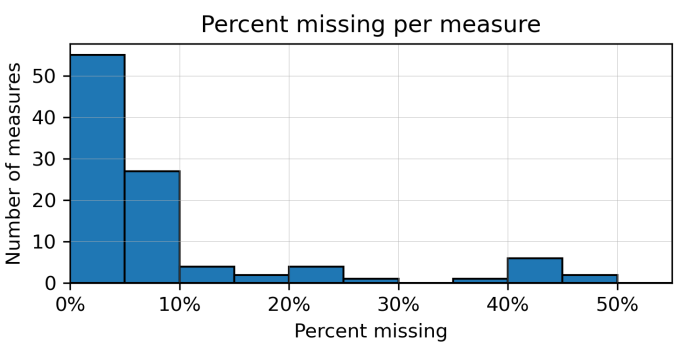

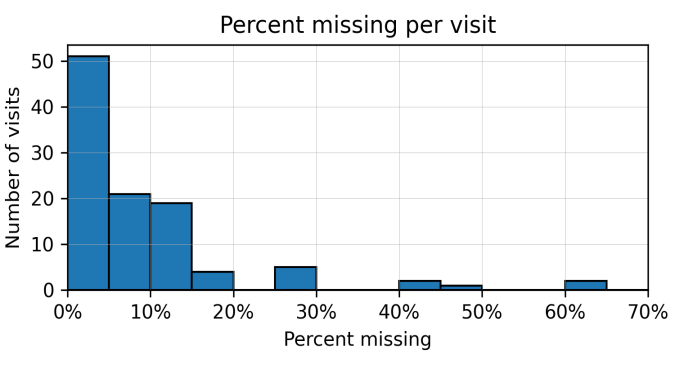
**Supplementary Figure 1: Missing entries**. Histograms of the percent of missing entries, calculated as a percent for each measure (left) and for each subject-visit (right). Data shown are for the 102 measures analyzed in the “Correlations among Measures” section, for the 105 visits of the symptomatic gene-positive males.

Due to the nonnegligible number of missing entries in the data, we used a Bayesian imputation approach to estimate the correlation between each pair of measures. Specifically, we used the following multivariate normal Bayesian model:

$$C \sim InverseWishart\left( S_{0},\nu_{0} \right)$$

$$\mu\sim MVN\left( m_{0},\Sigma_{0} \right)$$

$$Y_{i.t} \sim MVN(\mu,C)$$

where $C$ is the (unknown) $J\times J$ matrix of covariances and $J=102$ is the number of measures, $\mu$ is the (unknown) $J$-length vector of means, and $Y_{i.t}$ represents the $J$-length vector of measures for subject $i$ at time point $t$. For each $i$ and $t$, often only a subset of measures is observed, and the rest are missing. Letting $Y$ denote the complete data and letting $Y^{\text{obs}}$ denote the observed data entries, we compute the posterior distribution of $C, \mu, Y$ given $Y^{\text{obs}}$ by using a Gibbs sampler to update each of $C$, $\mu$, and $Y$, respectively, from their full conditional distributions. We use this Markov chain Monte Carlo sampler to approximate the posterior mean of $C$ and then we standardize it to obtain the resulting correlation matrix; this yields our estimate of the correlation matrix among measures as shown in Supplementary Figure 4. For the prior parameters, we use the following non-informative choices: $S_{0}=\varepsilon I$ for the scale matrix, $\nu_{0}=J$ for the degrees of freedom, $\mu_{0}=0$ for the prior mean, and $\Sigma_{0}=\left( 1/\varepsilon\right)I$ for the prior covariance, where $I$ denotes the $J\times J$ identity matrix and $\varepsilon={10}^{-8}$.

**Details on the Symptom Trajectory Model**

As we described in the main text, we applied Bayesian inference to fit our symptom trajectory model to observations on 87 participants (*n* = 29 symptomatic gene-positive, *n* = 7 pre-symptomatic gene-positive, and *n* = 51 gene-negative males) at different points of their symptom trajectories. Applying Bayesian inference requires specifying prior distributions on each of the parameters of inference. Below, we detail how we configured these prior distributions for each of the parameters in the model. Note that some of these priors are configured hierarchically, capturing known relationships between various parameters and data.

We model each gene positive individual’s age of onset as depending on their observed SVA repeat size. Specifically, we set

$$\text{Onse}\text{t}_{\text{i}} \sim\text{Normal}\left( \mu_{\text{onset}}+\beta_{\text{onset}}\text{ RepeatSize}_{i},\sigma_{\text{onset}} \right)$$

with priors on these parameters given by

$$\mu_{\text{onset}} \sim\text{Normal}\left( 40, 20 \right)$$

$$\sigma_{\text{onset}} \sim\text{InvGamma}\left( 1.5, 5 \right)$$

$$\beta_{\text{onset}} \sim\text{Normal}\left( 0, 10 \right).$$

As in the main text, Normal$\left( \mu,\sigma\right)$ denotes a normal distribution with mean $\mu$ and standard deviation $\sigma$. Moreover, we model each gene-positive subject’s reported age of onset as a mixture of two components: with probability 0.995,

$$\text{ReportedOnset}_{i}=\text{Onset}_{i}+\text{Weibull}\left( 2,\theta/\sqrt{\text{Rate}_{i}} \right),$$

and otherwise (that is, with probability 0.005),

$$\text{ReportedOnset}_{i} \sim\text{Uniform}\left( 0, 100 \right),$$

with a prior of $\theta\sim\text{Exponential}\left( 1 \right)$ on $\theta$. The predominant component is formulated as Weibull distribution such that each participant’s instantaneous rate of reporting onset is exactly proportional to their progression summary (the same quantity shown in Fig. 2B). The small uniform component assigns a small probability *a priori* to a participant’s reported onset being independent of their estimated onset. This makes the model robust to outliers due to potentially misattributed or misremembered reported ages of onset.

Note that for those gene-positive participants who have not yet reported an age of onset, their information contributes the model’s likelihood by conditioning on the fact that their reported age of onset exceeds their maximum observed age through the duration of the study. For each gene-positive participant’s rate of progression, we used independent

$$\text{Rate}_{i} \sim\text{Beta}\left( 2, 2 \right)$$

priors on their rate of progression, thus ensuring that their rates of progression are in [0,1] with a mean of 0.5 a priori. For each categorical measure, we assumed the priors

$$\beta_{j1} \sim\text{Normal}\left( 0, 5 \right)$$

$$\beta_{j2} \sim\text{Normal}\left( 0, 5 \right)$$

$$\alpha_{jk} \sim\text{Normal}\left( 0, 10 \right)$$

with the added restriction that $\alpha_{j1}<\alpha_{j2}<\cdots<\alpha_{jK}$. For each positive real-valued and non-negative integer measure, we assumed the same priors on $\beta_{j1}$ and $\beta_{j2}$ along with

$$\alpha_{j} \sim\text{Normal}\left( 0, 100 \right).$$

For the real-valued measures, we also assumed the prior

$$\sigma_{j} \sim\text{Exponential}\left( 1 \right).$$

The posterior distribution over the unknown model parameters was obtained by combining these prior distributions with the likelihood defined by the equations in the Symptom Trajectory Model section of the Materials and Methods.


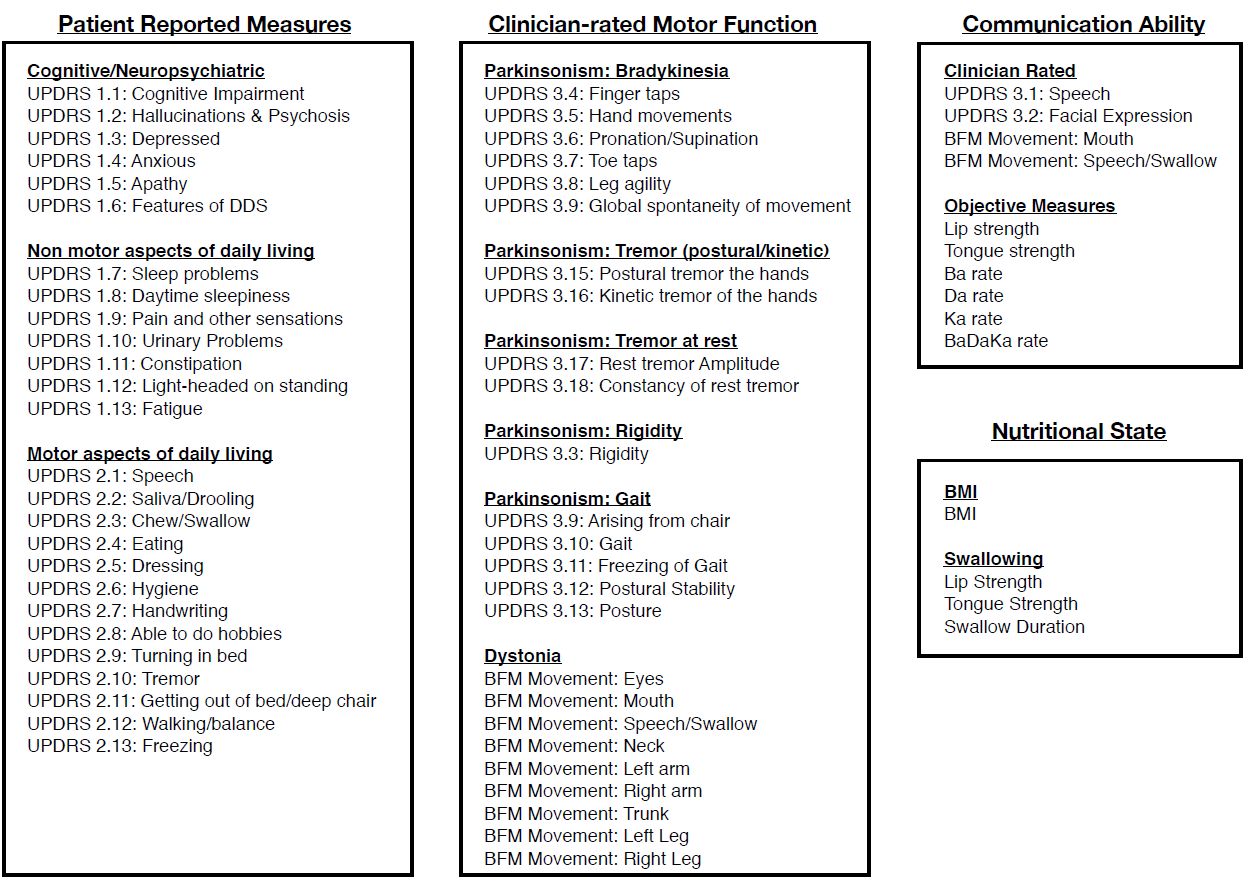


**Supplementary Figure 2.** Categories of measures used for rate heterogeneity analysis.


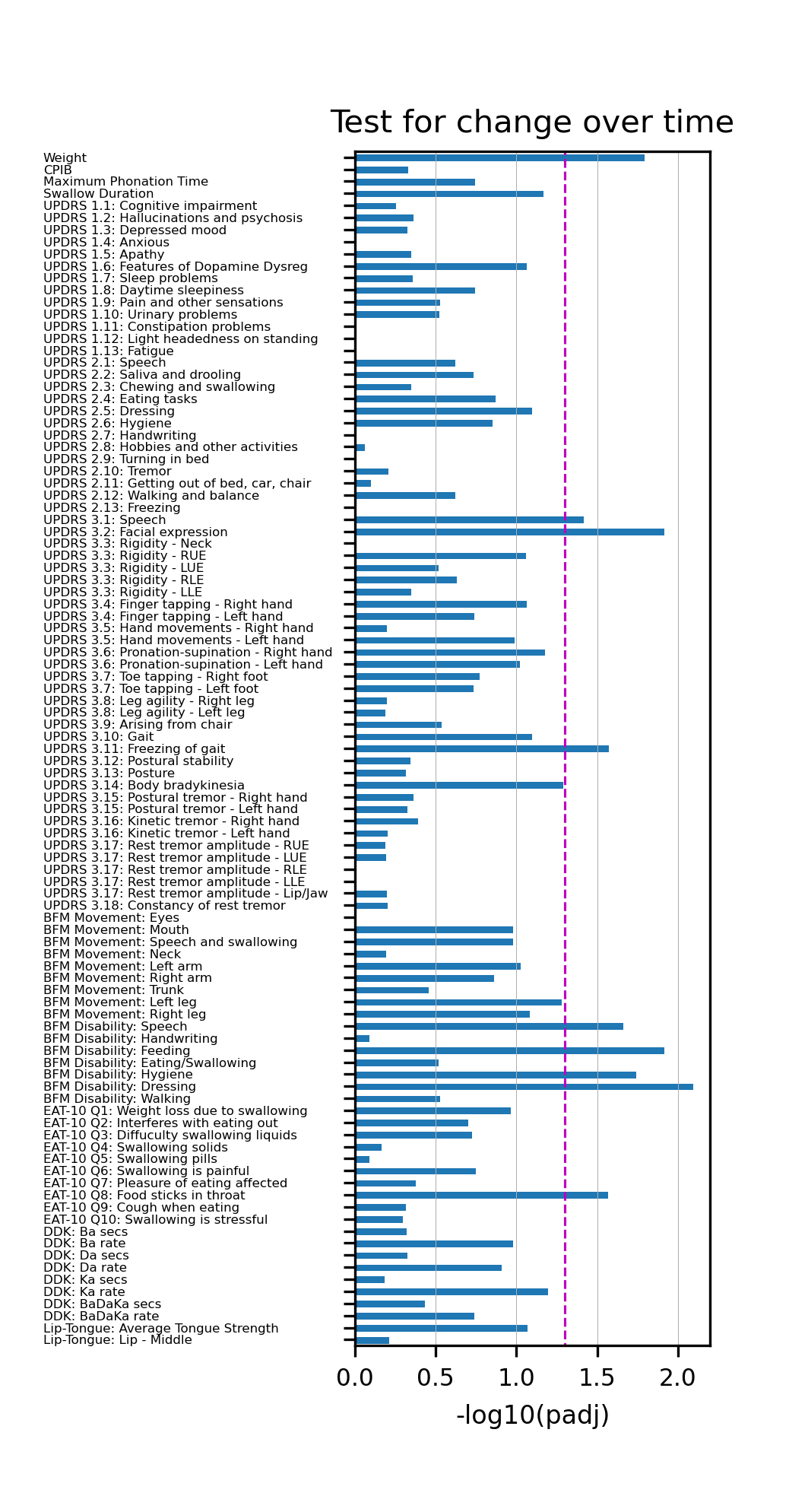


**Supplementary Figure 3: Hypothesis tests for change in each measure over time for symptomatic gene-positive males.** The length of each bar is -log_10_­(BH-adjusted p-value). The dotted line shows the significance threshold, controlling false discovery rate at 0.05 using the Benjamini-Hochberg (BH) procedure.


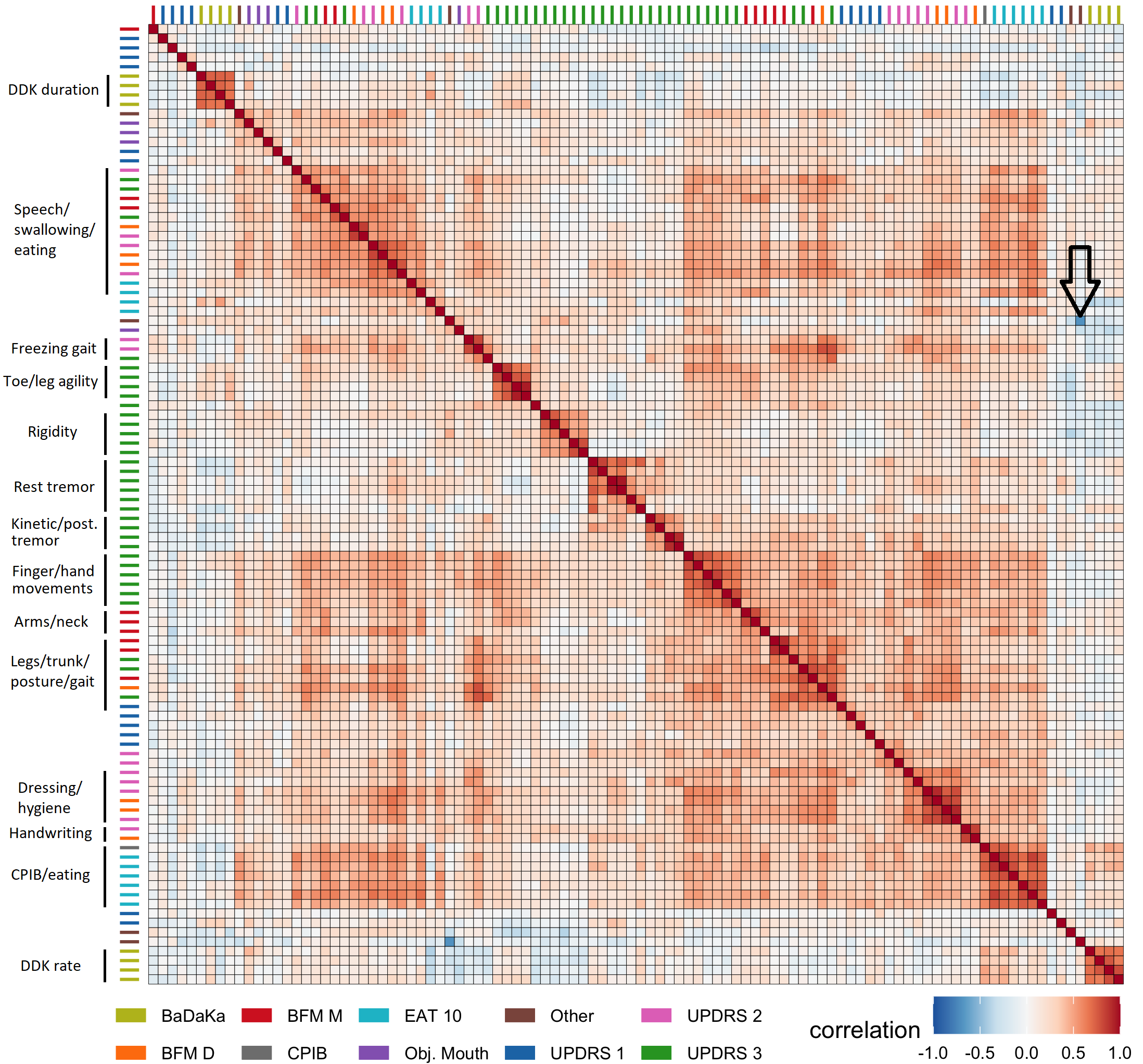


**Supplementary Figure 4: Correlation matrix among measure items.** The measures are ordered according to the dendrogram computed using hierarchical clustering in Supplementary Figure 5. The colors of bars on the left margin indicate the collection of measures to which each item belongs. The labels on the left margin indicate manually annotated clusters based on the dendrogram in Supplementary Figure 5. The one entry with a noticeably strong negative correlation, indicated by an arrow, is for repeat size and reported age at onset; also see Supplementary Figure 7.


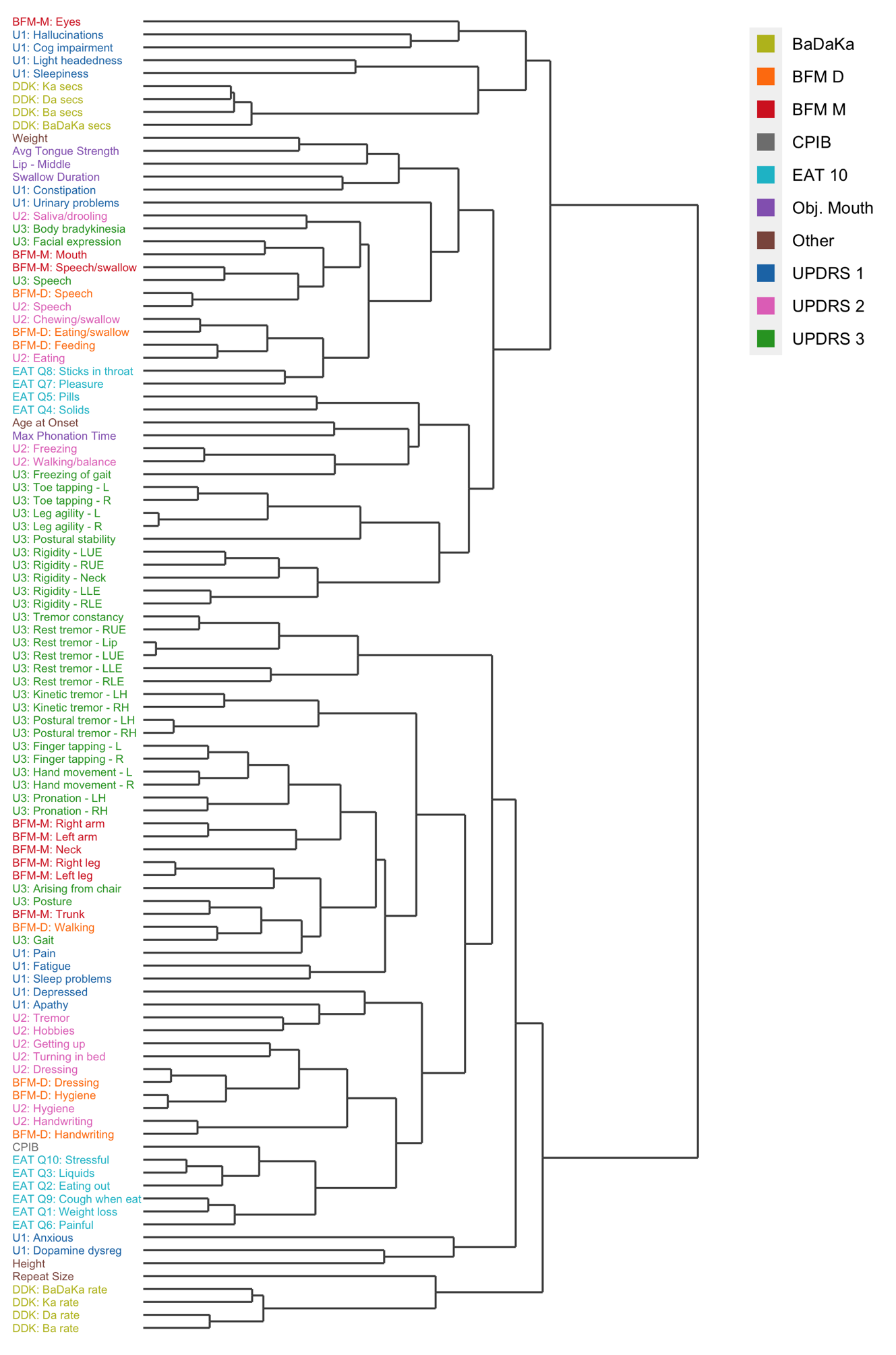


**Supplementary Figure 5: Dendrogram of measure items**. Computed based on hierarchical agglomerative clustering using the estimated correlation between each pair of individual measures.


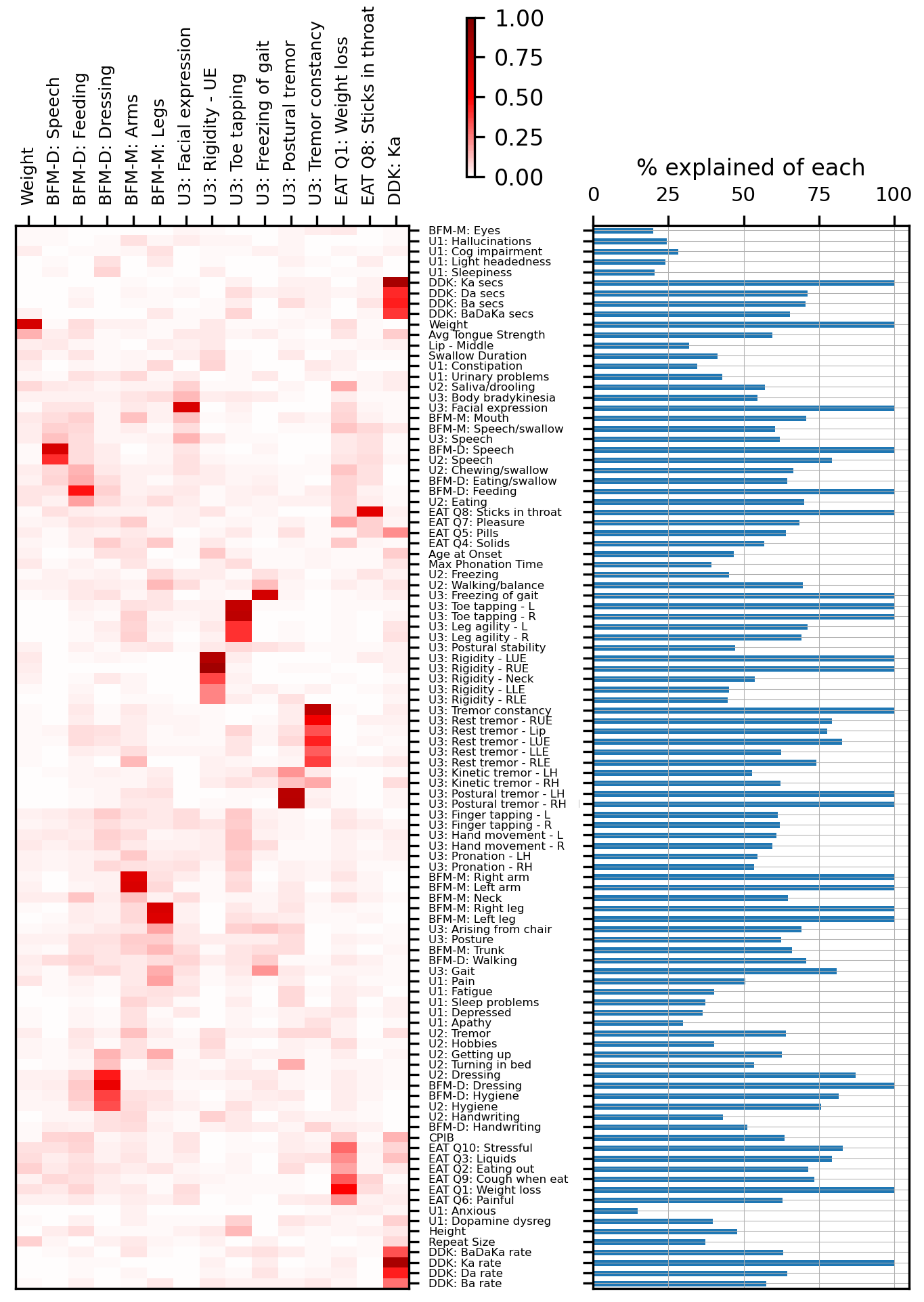


**Supplementary Figure 6.** Percent of each measure’s variance explained by the minimal battery (right), and how this divides up into the percent explained by each member of the minimal battery (left). The measures are ordered according to the dendrogram in Supplementary Figure 5.


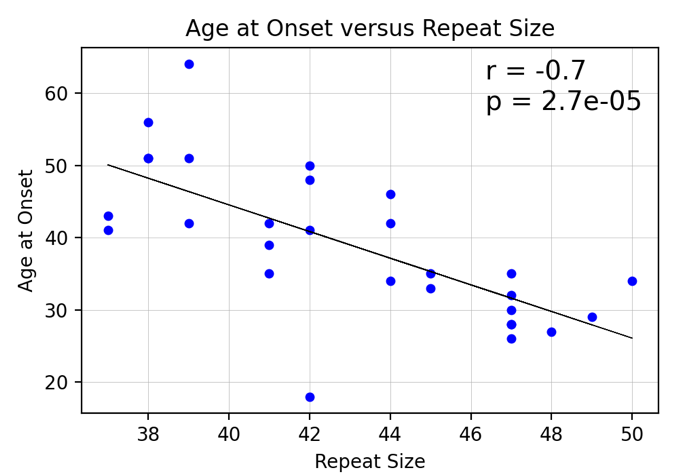


**Supplementary Figure 7.** Age at onset vs. repeat size. Age of onset is inversely associated with repeat size. Data shown for the symptomatic gene-positive males (n=29).


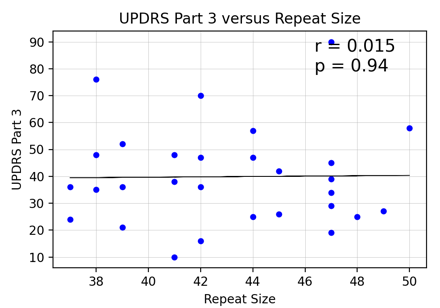

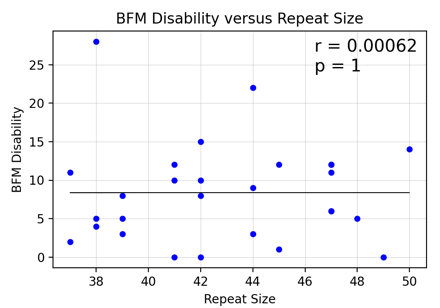

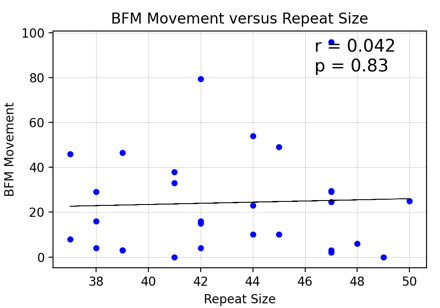


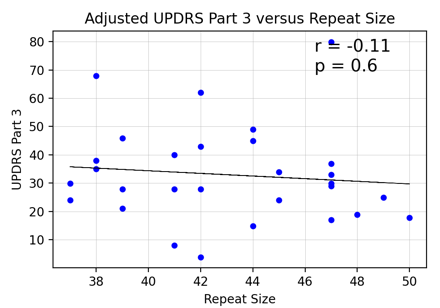

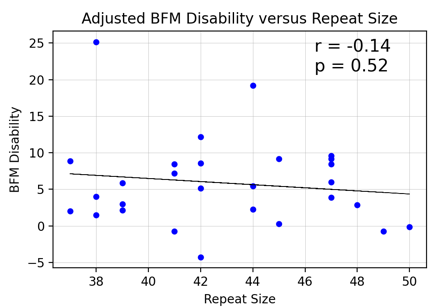

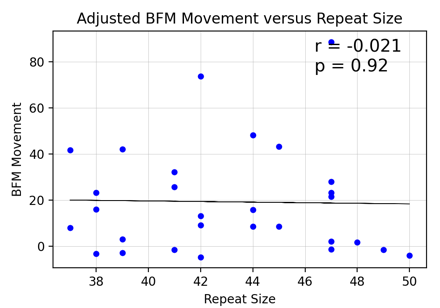


**Supplementary Figure 8. Disease severity measures and SVA repeat size.** Key disease severity measures do not exhibit a significant association with SVA repeat size. Data shown are severity measures at enrollment for the symptomatic gene-positive males (*n*=29). For BFM Disability, *n*=28 since one subject is missing this scale at enrollment.


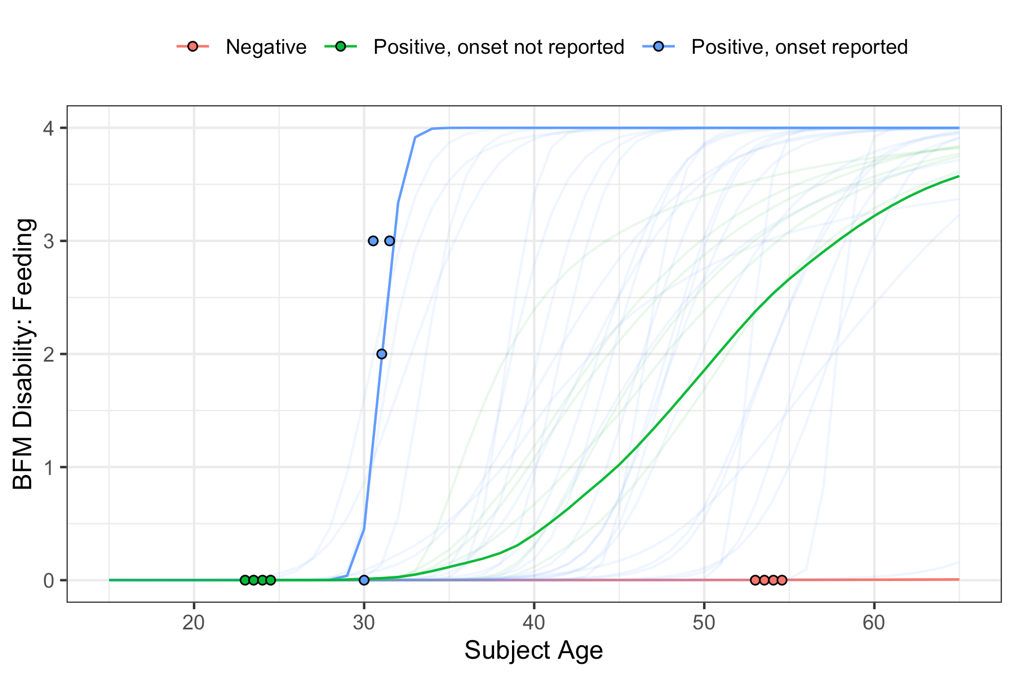


**Supplementary Figure 9.** **Illustration of extrapolated trend lines for BFM Disability: Feeding.** Lines depicting the estimated trend for all participants. Lines depicting the estimated trend for “BFM Disability: Feeding” for all participants. Three individuals—one gene-negative (red), one symptomatic gene-positive (blue), and one pre-symptomatic gene-positive (green)—are bolded, with their observed measurements shown as correspondingly colored points. This analysis involves *n*=29 symptomatic gene-positive, *n*=7 pre-symptomatic gene-positive, and *n*=51 gene-negative subjects.


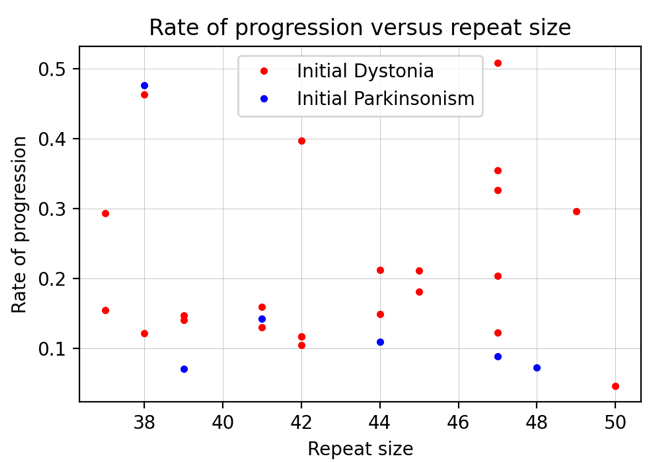

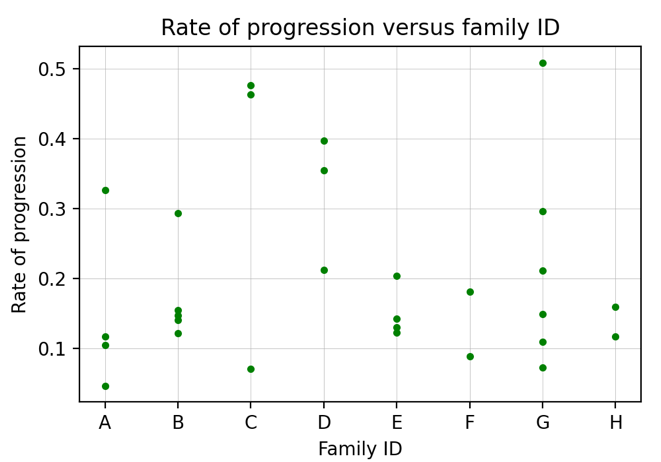


**Supplementary Figure 10. Disease comparison vs. repeat size or genetic background**. Estimated rate of progression does not appear to be associated with repeat size, initial symptom, or family ID. Data shown for symptomatic gene-positive males (*n*=29).


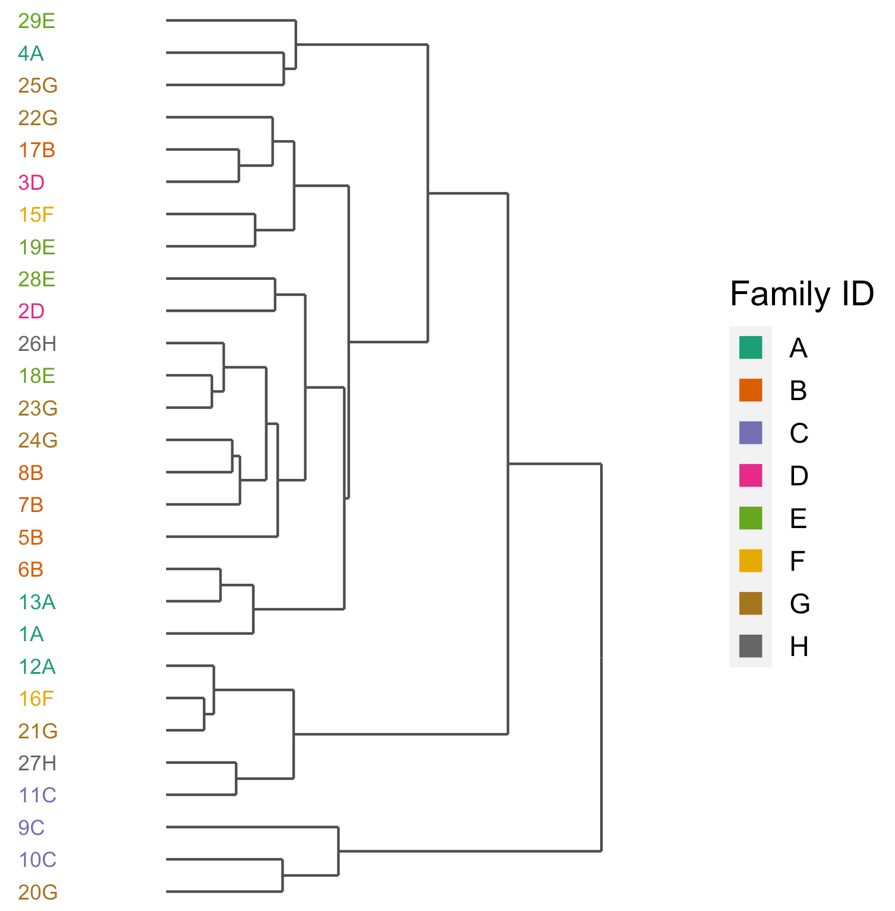


**Supplementary Figure 11:** Dendrogram based on hierarchical clustering of subjects according to their estimated rates of progression along the 13 categories in the rate heterogeneity analysis.


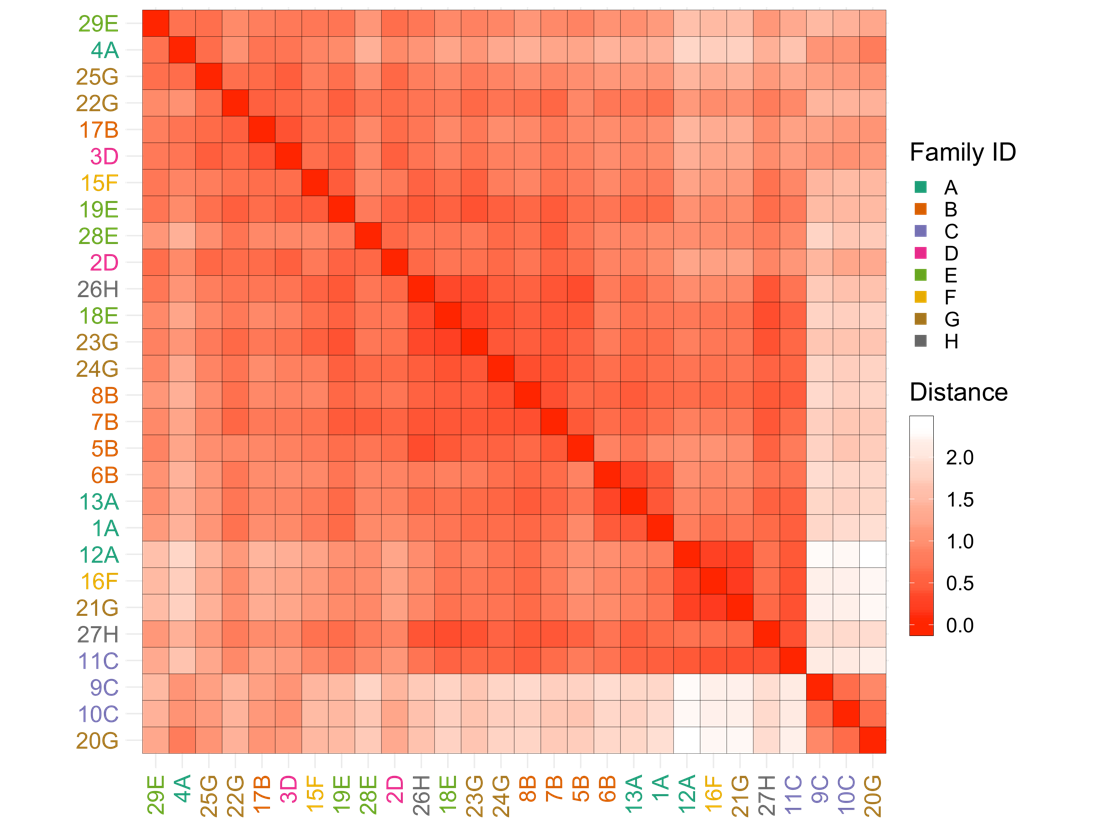


**Supplementary Figure 12**: Distance between each pair of subjects’ estimated rates of progression along the 13 categories that were pre-defined and shown in Supplementary Figure 3. Subjects are ordered according to the dendrogram in Supplementary Figure 11.
